# Genomic Evolution Strategy in SARS-CoV-2 Lineage B: Coevolution of *Cis* Elements

**DOI:** 10.3390/cimb46060344

**Published:** 2024-06-09

**Authors:** Yahaira de J. Tamayo-Ordóñez, Ninfa M. Rosas-García, Francisco A. Tamayo-Ordoñez, Benjamín A. Ayil-Gutiérrez, Juan M. Bello-López, Gerardo de J. Sosa-Santillán, Erika Acosta-Cruz, Francisco Anguebes-Franseschi, Siprian Damas-Damas, Angel V. Domínguez-May, Atl Victor Córdova-Quiroz, María Concepción Tamayo-Ordóñez

**Affiliations:** 1 Laboratorio de Biotecnología Ambiental del Centro de Biotecnología Genómica, Instituto Politécnico Nacional, Reynosa 88710, Tamps, Mexico; yahairatamayo@uadec.edu.mx; 2 Facultad de Química, Universidad Autónoma del Carmen, Calle 56 N. 4, Av. Concordia Col. Benito Juárez, Ciudad del Carmen 24180, Campeche, Mexico; ftamayo@pampano.unacar.mx (F.A.T.-O.); fanguebes@pampano.unacar.mx (F.A.-F.); sdamas@pampano.unacar.mx (S.D.-D.); acordova@delfin.unacar.mx (A.V.C.-Q.); 3 CONAHCYT—Centro de Biotecnología Genómica, Instituto Politécnico Nacional, Biotecnología Vegetal, Reynosa 88710, Tamps, Mexico; bayil@ipn.mx; 4 División de Investigación, Hospital Juárez de México, Ciudad de México 07760, Mexico; juanmanuelbello81@hotmail.com; 5 Laboratorio de Microbiología y Biosíntesis, Departamento de Biotecnología, Facultad de Ciencias Químicas, Universidad Autónoma de Coahuila, Saltillo 25280, Coahuila, Mexico; gdejsosa@uadec.edu.mx; 6 Laboratorio de Microbiología Molecular, Departamento de Biotecnología, Facultad de Ciencias Químicas, Universidad Autónoma de Coahuila, Saltillo 25280, Coahuila, Mexico; erika.acosta@uadec.edu.mx; 7 TecNM, Instituto Tecnológico Superior del Sur del Estado de Yucatán, Road Muna-Felipe Carrillo Puerto, Stretch Oxkutzcab-Akil Km 41+400, Oxkutzcab 97880, Yucatán, Mexico; adominguez@suryucatan.tecnm.mx; 8 Laboratorio de Ingeniería Genética, Departamento de Biotecnología, Facultad de Ciencias Químicas, Universidad Autónoma de Coahuila, Saltillo 25280, Coahuila, Mexico

**Keywords:** SARS-CoV-2, RNA structures, *cis* elements, coevolution

## Abstract

In the SARS-CoV-2 lineage, RNA elements essential for its viral life cycle, including genome replication and gene expression, have been identified. Still, the precise structures and functions of these RNA regions in coronaviruses remain poorly understood. This lack of knowledge points out the need for further research to better understand these crucial aspects of viral biology and, in time, prepare for future outbreaks. In this research, the in silico analysis of the *cis* RNA structures that act in the alpha-, beta-, gamma-, and deltacoronavirus genera has provided a detailed view of the presence and adaptation of the structures of these elements in coronaviruses. The results emphasize the importance of these *cis* elements in viral biology and their variability between different viral variants. Some coronavirus variants in some groups, depending on the *cis* element (stem-loop1 and -2; pseudoknot stem-loop1 and -2, and s2m), exhibited functional adaptation. Additionally, the conformation flexibility of the s2m element in the SARS variants was determined, suggesting a coevolution of this element in this viral group. The variability in secondary structures suggests genomic adaptations that may be related to replication processes, genetic regulation, as well as the specific pathogenicity of each variant. The results suggest that RNA structures in coronaviruses can adapt and evolve toward different viral variants, which has important implications for viral adaptation, pathogenicity, and future therapeutic strategies.

## 1. Introduction

SARS-CoV-2 is a member of the order Nidovirales, the family Coronaviridae, and the genus betacoronavirus lineage B [1]. Similar viruses, such as SARS coronavirus (SARS-CoV) and Middle East respiratory syndrome coronavirus (MERS), caused high mortality rates among infected people during the SARS and MERS outbreaks in 2002 and 2012, respectively [1]. The presence of *cis* elements of RNA in the SARS-CoV-2 lineage and their conservation in the taxonomic genera of coronaviruses have been detected in the genome. RNA elements acting in *cis* are essential for the viral life cycle of coronaviruses, i.e., RNA genome replication, viral gene expression, and genome packaging [2]. However, little information is available on the structures, functions, and interactions of these RNA regions in the coronavirus genome.

Members of the Coronaviridae virus family are single-stranded enveloped RNA viruses with positive polarity and large genomes of ~30 kilobases. These viruses contain a similar genomic RNA (gRNA) composition, including two open reading frames (ORF1a and ORF1b) encoding RNA-dependent RNA polymerase (RdRp) and non-structural proteins [3]. Likewise, they have the ability to produce subgenomic RNA (sgRNA), which serves as a template for the translation of the structural spike (S), membrane (M), virion envelope (E), and nucleocapsid proteins (N) [4]. The last of these proteins (N) forms ribonucleoprotein particles with RNAg, called the nucleocapsid complex, which is surrounded by a membrane consisting of proteins S, E, and M during the maturation process of the virion. The genomes of Coronaviridae viruses contain multiple structurally conserved elements within the 5′ and 3′ untranslated regions (UTRs) that have been suggested to play roles in viral gRNA and sgRNA replication. These elements acting in cis include three stem-loops (SL1, SL2, and SL3) within the 5′-UTR, as well as a bulging stem-loop (BSL), a pseudoknot stem-loop (PK), and a hypervariable region (HVR) within the 3′-UTR [1].

In coronaviruses, it has been described that during replication and transcription, the protein complex recognizes RNA elements that act in specific *cis* regions located mainly at the terminal ends (3′UTR, 5′UTR) and cover largely (but not exclusively) non-coding regions [5,6]. Additional elements acting in *cis* are located in internal positions and include transcription regulatory sequences (TRS) as well as specific RNA signals required for genome packaging into virions [7,8]. Another important structural element of RNA is located in the overlapping region of the reading frame (ORF1a). This complex pseudoknot structure (PK) mediates a ribosomal frame change event and thus controls the expression of the second longest length ORF in the coronavirus genome RNA (ORF1ab) [9].

The mapping of elements acting in *cis*, described for the first time in mouse hepatitis virus (MHV) and bovine coronavirus (BCoV), identified up to four stem-loops (SL Ia, Ib, III, IV) at the 5′ end (UTR) of the genome. In members of the Coronaviridae family, a model of three highly conserved stem-loop structures, called SL1, SL2, and SL4 (stem-loop), has been described in the 5′-UTR region of the genome, comprising approximately 150 nt [10]. SL1 and SL2 of the 5′-UTR end are predicted to be conserved in all genera of the Coronaviridae family [3]. In betacoronavirus, it has been shown that the structure adopted by SL1 and SL2 5′-UTR is fundamental for viral replication [11,12]. In the MHV genome, it has been suggested that SL1 requires optimal stability adequate to establish long-range transient interactions (mediated by RNA/proteins) between the 5′- and 3′-UTR ends that may be necessary for genome replication and subgenomic RNA synthesis. Furthermore, reverse genetic studies confirmed that SL2 5′-UTR is required for MHV RNA synthesis [11,12]. Based on phylogenetic analyses, SL2 was proposed to be the most conserved secondary RNA structure in coronaviruses.

Likewise, available information suggests that *cis* elements close to the 5′-UTR end may be structurally flexible and may adopt alternative structures to regulate specific steps of viral RNA synthesis [13]. The biological importance of analyzing the structure of *cis* elements close to the 5′- and 3′-end lies in the fact that if a conformational change such as the formation of stem-loop hairpins occurs, the translation of the information could not be carried out correctly. Derived from this, the study of the conformations acquired at both ends (5′- and 3′-) becomes important since, in the future, through the knowledge provided by this type of research complemented with genetic engineering, we could block the replication and transcription of the virus, which could be used in target therapies against COVID-19.

Recently, two additional SLs called SL-V and SL-VI were identified in the nsp1 coding region of BCoV, with SL-VI being essential for RNA replication. Overall, the available information suggests a model in which regions of the genome close to UTR-5′ of alpha- and betacoronavirus contain four major RNA structural elements: SL1, SL2, SL4, and SL5 [10,14]. The conservation of the structural elements of the SL1, SL2, SL4, and SL5abc RNA suggests important functions for these structures in the coronavirus life cycle. Possible SL5 substructures in alpha- and betacoronavirus may be located within the coding region of nsp1 (formerly referred to as SL IV, V, VI, and VII) or extend to ORF1a. Depending on the lineage studied, conserved structures can be identified in the SL5 hairpin substructures [10].

The first studies of *cis* elements in the 3′-UTR were performed in the betacoronavirus genus, which found elements necessary for replication, changes in the ribosomal framework, and viral messenger RNA packaging (mRNA) [15]. Within these, there is a 68 nts bulging stem-loop (BSL) that lies immediately downstream of the stop codon of the N gene and was shown to be necessary for MHV RNA replication [16]. The second essential RNA structure is a classical 54 nt BCoV fork-like pseudoknot (PK) structure. The third secondary structure of RNA is a long multibranched stem-loop (SL) structure downstream of the pseudoknot [17].

RNA therapy is a rapidly developing field and has significant potential in fighting against SARS-CoV-2. Studies showed that the therapeutic siRNA, which directly targeted the leader sequence of SARS, could also inhibit the viral replication in Vero E6 cells, suggesting that the UTR of SARS may serve as a potential target of RNAi. Since the secondary structures of the 5′UTR of SARS-CoV and SARS-CoV-2 are remarkably alike, these specific region sequences can also be the target for the treatment of COVID-19 using RNA therapeutics.

With great progress in RNA therapy, it still has some limitations in practice. Due to its poor stability, RNA can be easily degraded, making the biological half-life too short. The low efficiency of cellular uptake makes it difficult for the drugs to get into the target cells. Furthermore, RNA itself is potentially immunogenic and likely to have off-target effects. For drugs used in therapy or for genetic engineering technologies to be successful, it is necessary to know the biological nature of the target.

In this research, the role of key elements, stem-loop1 and -2, and pseudoknot stem-loop1 and -2, and s2m, in the genome of alpha-, beta-, gamma-, and deltacoronavirus has been identified and explored. These *cis* elements are crucial in the interaction with viral host proteins and other RNA factors; they are involved in the regulation of processes such as translation and replication of the viral genome, as well as virus encapsulation. Understanding their evolution and function can help in formulating strategies to control pandemics. In this study, the structural and functional coevolution of these elements in coronavirus genera, including the B lineage of SARS-CoV-2, is analyzed with the aim of using them as genetic biomarkers to understand the origin and divergence of new variants, such as SARS, OMICRON, ERIS, and PIROLA, and to develop possible therapies.

## 2. Materials and Methods

### 2.1. Identification of Cis Elements in the Genome of Human Pathogenic Coronaviruses

Four phylogenetically distinct groups and seven human coronavirus variants (hCoVs) were included in this study. The genomes of coronavirus variants were human alphacoronavirus 229E (HCoVs-229E, NC_002645), human alphacoronavirus NL63 (HCoVs-NL63, NC_005831.2), human betacoronavirus OC43 (HCoVs-OC43, NC_006213.1), human betacoronavirus HKU1 (HCoVs-HKU1, NC_006577.2), Middle East respiratory syndrome-related coronavirus (MERS-CoV, NC_19843.1), Middle East respiratory syndrome-related coronavirus—England (MERS-CoV-Eng, NC_038294), severe acute respiratory syndrome-related coronavirus (SARS-CoV, KY352407.1), severe acute respiratory syndrome-related coronavirus 2 (SARS-CoV-2, NC_045512), severe acute respiratory syndrome-related coronavirus Tor2 (SARS-CoV-Tor2, NC_04718), duck avian coronavirus (AcCoV-Duck, NC_048214), turkey avian coronavirus (AcCoV-Turkey, NC_010800), porcine deltacoronavirus HKU15 (HKU15, MW685622.1), and sparrow deltacoronavirus HKU17 (HKU17, MG812375) released in the NCBI virus database (National Center for Biotechnology Information; https://www.ncbi.nlm.nih.gov/labs/virus/vssi/#/, accessed on 5 June 2023). These accessions correspond to complete genomes of lineage B origin of SARS-CoV-2. These variants were considered in each of the alpha-, beta-, gamma-, and deltacoronavirus genera because they allow us to know the evolutionary trajectory of the origin of SARS-CoV-2. It is worth mentioning that there is limited free information of genomes of these coronavirus genera; types of coronavirus hosts of humans and mammals were selected (except for deltacoronavirus) (see Table 1). To identify the *cis* elements in the genome of the coronavirus variants, an analysis of sequence similarity, position, and genomic location was carried out using the ClustalW bioinformatics tool from BioEdit, and MEGA was used as NCBI Reference Sequence NC_045512.2 (https://www.ncbi.nlm.nih.gov/labs/virus/vssi/#/) (see Figure S1).

### 2.2. Secondary Structures of Cis and ORF Elements of Coronavirus Genomes

For the analysis of the *cis* elements in the viral genome, structure prediction programs based on thermodynamic principles and exact dynamic programming algorithms were used to calculate ground states and base coincidence probabilities to predict the most stable secondary structures of the virus genomes, which can provide crucial information to understand their function and biology.

In this study, to know the behavior of *cis* elements in the coronavirus genome (HCoVs-229E, HCoVs-NL63, MERS-CoV-Eng, SARS-CoV, SARS-CoV-2, AcCoV-Duck, Ac-CoV-Turkey, HKU15, HKU17) the prediction of the secondary structures of the *cis* elements was carried out from three approaches: the first was individually (stem-loop1 and -2, pseudoknot stem-loop1 and -2, and s2m); the second formed a genetic unit (stem-loop1, 2 pseudoknot stem-loop1, 2); and in the third involved its conformation in the genomic region where they are located, wherein stem-loop1 and -2 were analyzed in ORF1ab, along with the stem-loop1 and -2 pseudoknot in ORF1a and the s2m in the 3′-UTR. In the open reading frames, the greatest length that the web server respects is covered.

For the prediction of the secondary structures of the *cis* elements (stem-loop1 and -2 and pseudoknot stem-loop1 and -2, and s2m), the RNAfold was used, and the functionality of the structures was analyzed with the Structure Conservation Analysis of the ViennaRNA Web Services (http://rna.tbi.univie.ac.at/, accessed on 5 February 2024). The RNAfold is based on partition function calculations and suggests different thermodynamic prediction models of minimum free energy (ΔG). Using the energy minimization algorithm, the structure that minimizes the overall free energy of the RNA structure was selected. The software identifies the secondary structure that has the lowest free energy, making it more thermodynamically stable. Within the parameters, the minimum free energy (MFE) and partition function algorithm, which calculates the partition function and the base pairing probability matrix, were adjusted. In addition to the minimum free energy (MFE) structure, the energy parameters used in the calculation have been measured under standard conditions and at 37 °C.

Structure Conservation Analysis calculates the conservation probability and score using SCI (structure conservation index), which is based on the consensus energy calculated by RNAfold, and the conservation score is evaluated by comparing the energies of the sequences under the constraint of being seen forced to fold into the structures of the other sequences for native energies. The percentage of structural similarity was through the p value (statistical measure of reliability of structural predictions). The length of the sequences was estimated by the comparative alignment for the RNA sequences of interest.

The conformation of the RNA of the ORF1ab, accessory ORF1a, and 3′UTR of the coronavirus variants was carried out by using the RNAstructure software of Web Servers for RNA Secondary Structure Prediction (https://rna.urmc.rochester.edu/RNAstructureWeb/Servers/Predict1/Predict1.html, accessed on 1 March 2024). The server provides a secondary structure by combining four prediction algorithms: (1) calculation of a partition function, (2) prediction of a minimum free energy (MFE) structure, (3) simulation of the structure with the maximum expected accuracy, and (4) pseudoknot prediction.

### 2.3. Mutational Distance and Evolutionary Relationships of Cis Elements in Coronaviruses

To understand how *cis* elements in coronavirus genera have evolved and diverged over time, the evolutionary and phylogenetic relationships between these *cis* elements were determined. This provides important information on the evolution and genetic variability of coronaviruses, which is relevant to understanding their epidemiology, pathogenicity, and evolutionary potential in the context of human diseases. In this sense, two explorations were carried out. The first was a sequence similarity analysis using molecular evolutionary genetics analysis, which allows for the reconstruction of the evolutionary history of coronavirus variants. And the construction of a haplotype network is also needed to determine the genetic variability and the relative frequency of mutations within the coronavirus genera.

The haplotype model was constructed on the Network v.4.6 software, using the median-joining method assuming an epsilon of 0 and a transversion/transition ratio of 1:2 [18]. The median-joining (MJ) method begins with the minimum spanning trees, all combined within a single (reticulate) network. Aiming at parsimony, we subsequently added a few consensus sequences (i.e., median vectors or Steiner points) of three mutually close sequences at a time. The median operation is basic to all fast MP heuristic algorithms (p-median) and is applied in a very restricted way to arrive at a single set of related data [19]. To identify genomic regions of interest and to understand the genetic variability of *cis* viral elements, a statistical analysis was performed to identify genetic associations, determining genetic diversity and phylogenetic relationships between different populations. A statistical analysis was included to identify genetic associations and determine genetic diversity and phylogenetic relationships between different virus genera. 

Evolutionary analyses were performed in MEGA11 [20], and evolutionary history was performed using the minimal evolution method. Sequence alignment was performed by ClustalW and the MEGA module to select the most appropriate evolutionary model for the dataset. The percentages of replicated clusters were calculated with a bootstrap of 1000 repetitions. Evolutionary distances were quantified by using the Poisson correction method based on the number of ribonucleotide substitutions per site. The dendrogram was constructed by using the Close-Neighbor-Interchange (CNI) algorithm.

## 3. Results

### 3.1. Identification and Organization of Cis Elements in Human Pathogenic Coronaviruses

We reported the exploration and identification of the *cis* elements (stem-loop1 and -2 and pseudoknot stem-loop1 and -2, and s2m) in the genome of four coronavirus genera, including the betacoronavirus lineage characterized by SARS-CoV variants. In general, all coronaviruses in this study have a ~30 kb length, comprising ORF1ab and ORF1 (HCoVs-229E, HCoVs-NL63, HCoVs-OC43, HCoVs-HKU1, MERS-CoV, MERS-CoV-Eng, SARS-CoV, SARS-CoV-2, SARS-CoV-Tor2, AcCoV-Duck, AcCoV-Turkey, HKU15, HKU17). In the open reading frame, the presence of the stem-loop1 and -2 and pseudoknot stem-loop1 and -2 were located. These sequences are delimited by the 5′-UTR and 3′-UTR regions, where the s2m is located.

**Figure 1:**
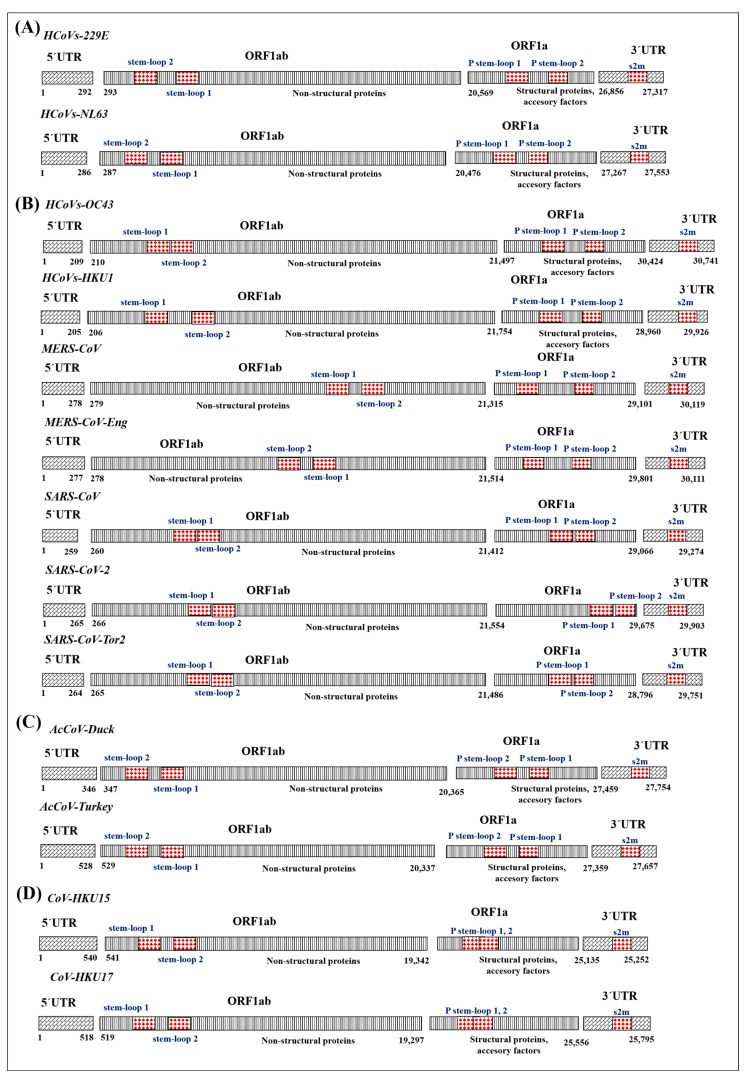
General scheme of organization of *cis* elements in alpha-, beta-, gamma-, and deltacoronavirus genomes. The 5′ and 3′ UTR terminal ends are illustrated, and the open reading frame (ORF) 1ab that encodes the polyprotein corresponding to non-structural proteins (Nsp) is located within the ORF of the stem-loop1 and -2; ORF1a represents the coding regions of structural and accessory proteins, as well as the *cis* elements (pseudoknot stem-loop1 and -2). The s2m is located in the 3′-UTR. The genome size of SARS-CoV-2 is ~30 kb in length. (**A**) Alphacoronavirus: HCoVs-229E/NP_002645; HCoVs-NL63/NC_005831. (**B**) Betacoronavirus: HCoV-OC43/NC_006213; HCoV-HKU1/NC_006577; MERS-CoV/NC_19843, MERS-CoV-Eng/NC_038294; SARS-CoV/KY352407; SARS-CoV-2/NC_04718. (**C**) Gammacoronavirus: AcCoV-Duck/NC_048214; Ac-CoV-Turkey/NC_010800. (**D**) Deltacoronavirus: HKU15/MW685622; HKU17/MG812375.

The genetic map indicated the presence of these *cis* elements, showing a variation in position, location, and length according to the genome of the viral variant. In the group of alphacoronavirus (HCoVs-229E, HCoVs-NL63) and gammacoronavirus (AcCoV-Duck, AcCoV-Turkey), an inversion in the characteristic position of stem-loop2 close to the 5′-UTR end was identified. The variants of betacoronaviruses showed two structural patterns of *cis* elements: the coronaviruses SARS-CoV, SARS-CoV-2, and SARS-CoV-Tor2 show the proximity of the stem-loop and pseudoknot stem-loop, and the genome of HCoVs-HKU1, MERS-CoV, and MERS-CoV-Eng display separation in the position of these *cis* elements. In the deltacoronaviruses HKU15 and HKU17, a short location of pseudoknot stem-loop is observed (Figure 1).

The observation of variations in position, location, and length of *cis* elements in the genome of different viral variants, as revealed in the genetic map, is an intriguing finding that highlights the diversity and adaptability of coronaviruses. These structural variations in the *cis* elements may have important implications for the biology and pathogenicity of these viruses, as well as their ability to evade the immune system and develop specific replication strategies.

In the alpha- and gammacoronavirus group, the identification of an inversion in the characteristic position of stem-loop2 near the 5′-UTR terminal suggests a specific adaptation of these viruses throughout their evolution. This inversion could have functional consequences on the regulation of gene expression and genome structure, which could influence viral replication and host interaction. In the case of betacoronaviruses, two distinct structural patterns of *cis* elements are observed. The proximity between the stem-loop and pseudoknot stem-loop in the SARS-CoV, SARS-CoV-2, and SARS-CoV-Tor2 coronaviruses could indicate an evolutionary convergence in the organization of these elements, suggesting its importance in the biology of human pathogenic betacoronaviruses. On the contrary, the separation in the position of these *cis* elements in the genomes of HCoVs-HKU1, MERS-CoV, and MERS-CoV-Eng illustrates the genomic plasticity of coronaviruses, which could be related to their ability to adapt to different hosts or environmental conditions. In deltacoronaviruses HKU15 and HKU17, the nearby location of the pseudoknot stem-loop is also a relevant finding. This points to a convergence in the organization of *cis* elements in these viruses, suggesting the importance of this specific structure in their biology and pathogenicity.

The results highlight the complex evolution of coronaviruses and the plasticity of their genomes. Variation in the organization of *cis* elements may be related to adaptation to different ecological niches and interaction with multiple host species over time to human transmission. Understanding these variations in the organization of *cis* elements is essential to obtain a better view of the biology and evolution of coronaviruses, as well as for the development of prevention and effective treatment strategies.

### 3.2. Diversity and Structural Functionality of Cis Elements in Coronaviruses

In silico analysis of the conservation of *cis*-acting RNA structures in the alpha-, beta-, gamma-, and deltacoronavirus genera provides interesting insight into the diversity and functionality of these elements in coronaviruses. In this study, possible structural and functional features of key elements, including stem-loop1 and -2, pseudoknot stem-loop1 and -2, and s2m, were identified and explored.

**Figure 2:**
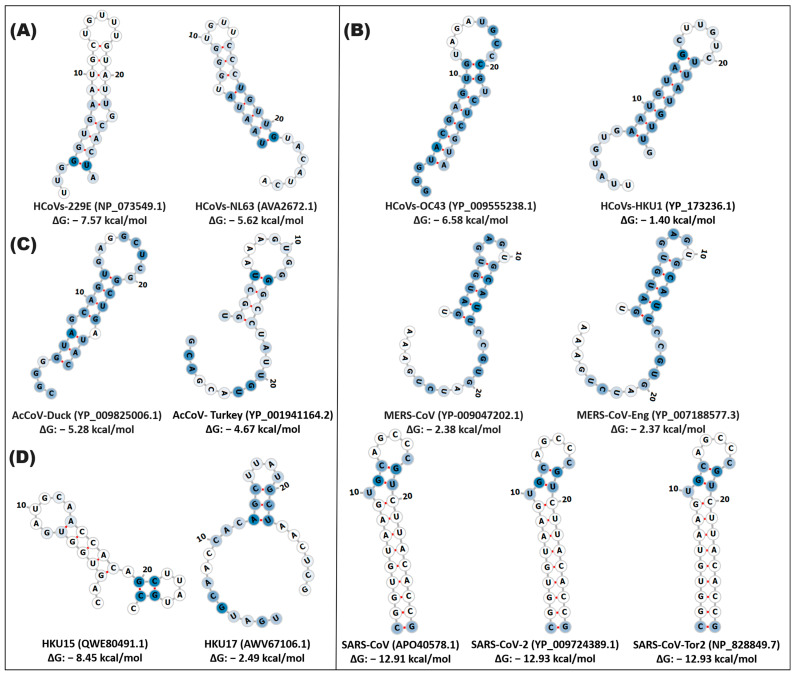
In silico secondary structure of stem-loop1 of alpha-, beta-, gamma-, and deltacoronaviruses. The prediction of the secondary structure of stem-loop1 was made on the Web Server RNAfold of the ViennaRNA. (**A**) alphacoronavirus; (**B**) betacoronavirus; (**C**) gammacoronavirus; (**D**) deltacoronavirus. In the highlighted panel, the types of coronaviruses close to SARS-CoV-2, which cause COVID-19, are grouped. The white and blue nucleotides indicate the MFE value of the ribonucleotides. The hydrogen bonds of the ribonucleotides are illustrated in red.

In the betacoronavirus genus, two groups are distinguished in the conformation of the secondary structure of stem-loop1. In particular, the group of MERS-CoV variants, MERS-CoV-Eng and SARS-CoV, SARS-CoV-2, and SARS-CoV-Tor2 exhibited structural and sequence conservation with a probability of a functional conformation of 98% and >75%, respectively, suggesting functional importance in the viral biology of these variants. MERS-CoV and MERS-CoV-Eng presented an 8rb stem and 6rb loop defined with a ΔG of −2.38, −2.37 kcal/mol, while SARS-CoV, SARS-CoV-2, and SARS-CoV-Tor2 contain a stem made up of 16rb and two loops of 5 and 7rb in the same structural position with a ΔG of −12.91, −12.93 kcal/mol (Figure 2 and Figure S1A, Tables S1 and S8).

**Figure 3:**
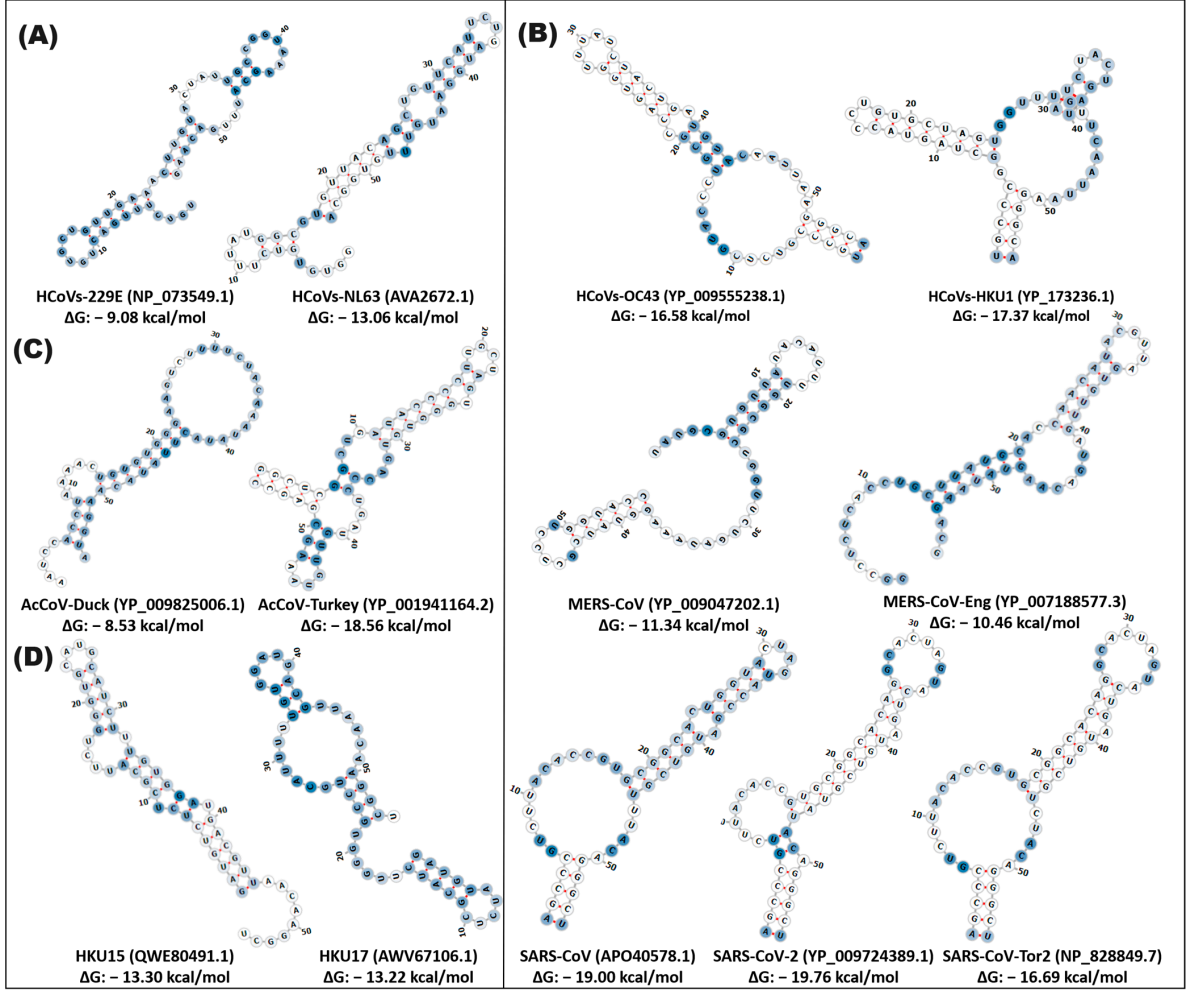
In silico secondary structure of stem-loop2 of alpha-, beta-, gamma-, and deltacoronaviruses. The prediction of the secondary structure of stem-loop2 was made by the Web Server RNAfold of the ViennaRNA. (**A**) alphacoronavirus; (**B**) betacoronavirus; (**C**) gammacoronavirus; (**D**) deltacoronavirus. In the highlighted panel, the types of coronaviruses close to SARS-CoV-2, which cause COVID-19, are grouped. The white and blue nucleotides indicate the MFE value of the ribonucleotides. The hydrogen bonds of the ribonucleotides are illustrated in red.

In contrast, the stem-loop2 RNA element exhibits a differential pattern in its structural conservation in coronavirus variants. Thus, the HCoVs-OC43 and HCoVs-HKU1 variants present sequence conservation with three key changes (position 10, 21, and 36) with a different structural conformation with a low probability of functionality (43%) and a ΔG of −16.58, −17.37 kcal/mol. In the case of the SARS variants, sequence conservation is observed with some specific changes (position 28, 31, 40, 50) and a similar secondary structure in which the formation of two loops and a defined stem can be distinguished. SARS-CoV and SARS-CoV-Tor2 share a 21rb loop (position 5–18, 45–51), while SARS-CoV-2 and SARS-CoV-Tor2 have an 11rb loop (position 26–36) at the end of the structure and an 8rb stem (position 1–4, 52–55), with a probability > 76% functionality with a ΔG of −19.00, −19.76, −16.69 kcal/mol (Figure 3 and Figure S1E, Tables S2 and S9).

**Figure 4:**
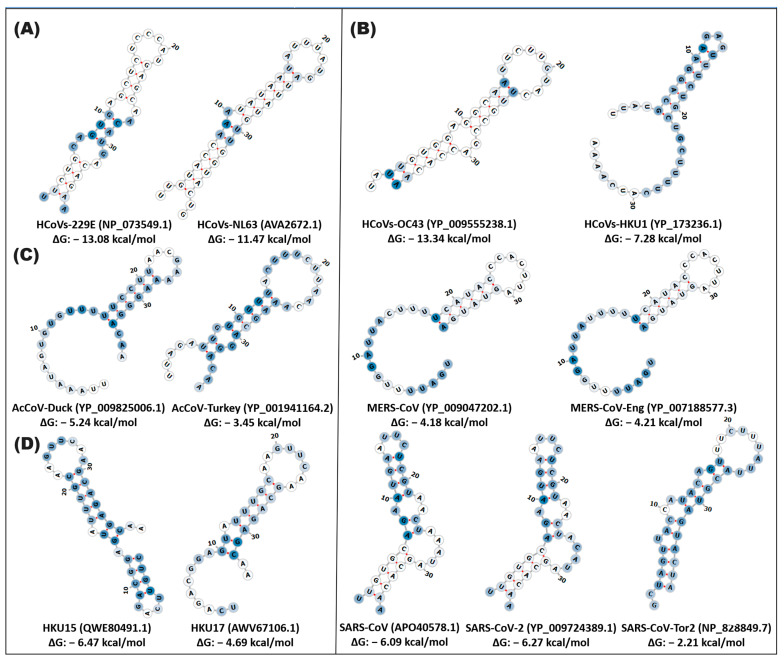
In silico secondary structure of pseudoknot stem-loop1 of alpha-, beta-, gamma-, and deltacoronaviruses. The prediction of the secondary structure of pseudoknot stem-loop1 was performed on the Web Server RNAfold of the ViennaRNA. (**A**) alphacoronavirus; (**B**) betacoronavirus; (**C**) gammacoronavirus; (**D**) deltacoronavirus. In silico secondary structure of stem-loop2 of alpha-, beta-, gamma-, and deltacoronaviruses. In the highlighted panel, the types of coronaviruses close to SARS-CoV-2, which cause COVID-19, are grouped. The white and blue nucleotides indicate the MFE value of the ribonucleotides. The hydrogen bonds of the ribonucleotides are illustrated in red.

In particular, the secondary conformation of the stem-loop1 pseudoknot conserves a structure and sequence in the MERS-CoV and MERS-CoV-Eng variables with the formation of a 10rb stem (position 18–22, 31–36) and a 9rb loop (position 23–31) with a similar ΔG (ΔG: −4.18, −4.21 kcal/mol). SARS-CoV and SARS-CoV-2 exhibit structures with an 8rb stem (position 2–5, 32–35) and three small loops (position 6–31) with a ΔG of −6.09, −6.27 kcal/mol. These betacoronavirus variants showed a low probability of functionality < 50% (Figure 4 and Figure S1C, Tables S3 and S10).

**Figure 5:**
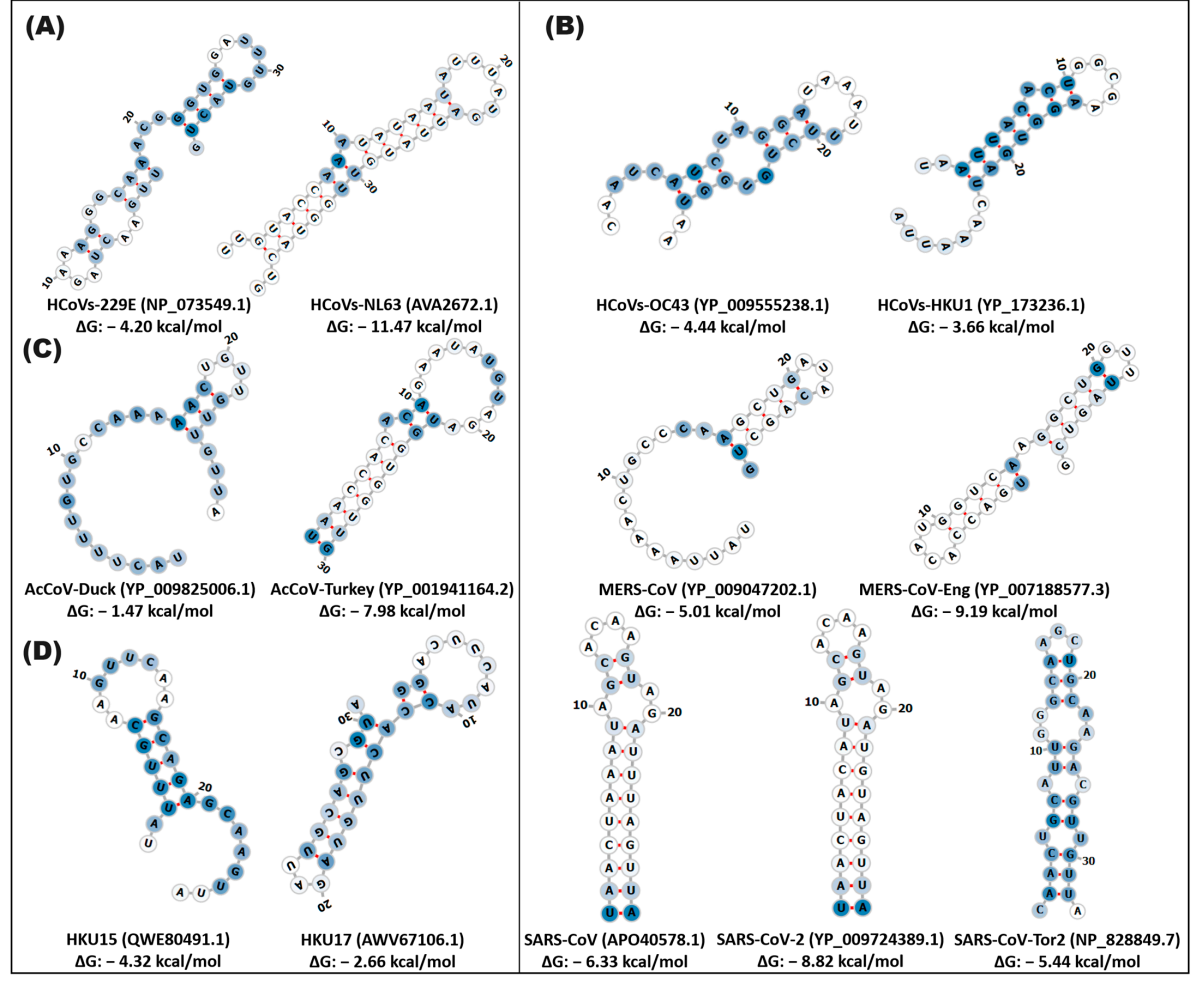
In silico secondary structure of pseudoknot stem-loop2 of alpha-, beta-, gamma-, and deltacoronaviruses. The prediction of the secondary structure of pseudoknot stem-loop2 was made on the Web Server RNAfold of the ViennaRNA. (**A**) alphacoronavirus; (**B**) betacoronavirus; (**C**) gammacoronavirus; (**D**) deltacoronavirus. In the highlighted panel, the types of coronaviruses close to SARS-CoV-2, which cause COVID-19, are grouped. The white and blue nucleotides indicate the MFE value of the ribonucleotides. The hydrogen bonds of the ribonucleotides are illustrated in red.

The stem-loop2 pseudoknot of the alpha-, beta-, gamma-, and deltacoronavirus variants stand out for secondary structures with a high degree of differences in conformation. With the exception of SARS-CoV and SARS-CoV-2, which present a similar structural conformation with only two ribonucleotide changes, where the formation of a 16rb stem (position 1 to 9, 22–29) and two loops of 7 and 6rb (position 9–21) with ΔG of −6.33, –8.82 kcal/mol with a low probability of functionality (20%) (Figure 5 and Figure S1D, Tables S4 and S11).

**Figure 6:**
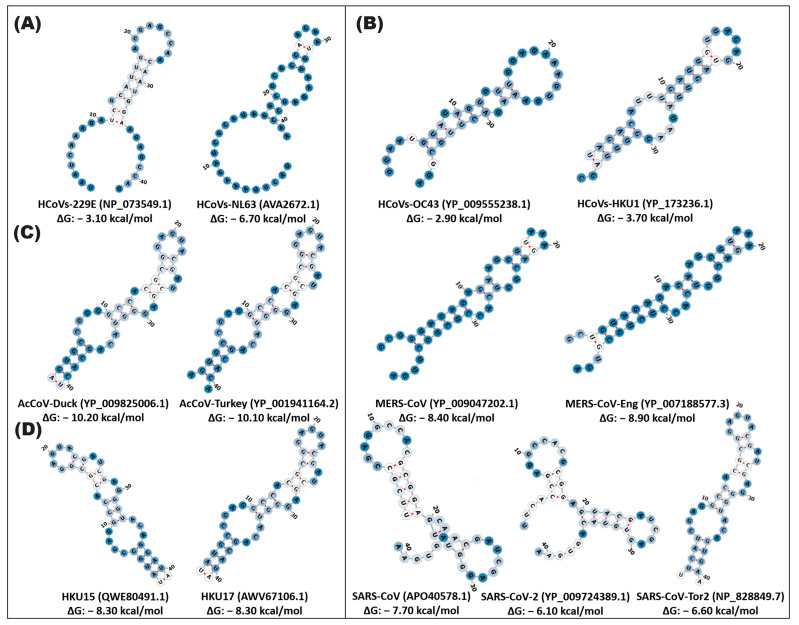
In silico secondary structure of s2m of alpha-, beta-, gamma-, and deltacoronavirus. The prediction of the secondary structure of s2m was made on the Web Server RNAfold of the ViennaRNA. (**A**) alphacoronavirus; (**B**) betacoronavirus; (**C**) gammacoronavirus; (**D**) deltacoronavirus. In the highlighted panel, the types of coronaviruses close to SARS-CoV-2, which cause COVID-19, are grouped. The white and blue nucleotides indicate the MFE value of the ribonucleotides. The hydrogen bonds of the ribonucleotides are illustrated in red.

The analysis of the in silico secondary structures of s2m in the alpha-, beta-, gamma-, and deltacoronaviruses genera provided valuable information on the flexibility and structural conservation of this element. In the beta- (MERS-CoV, MERS-CoV-Eng), gamma- (AcCoV-Duck, AcCoV-Turkey), and deltacoronavirus variants (DeltaCoV-HKU15, DeltaCoV-HKU17), a structural and sequence conformation with a probability of functionality is observed (>70%). MERS-CoV and MERS-CoV-Eng present a single change from U to G at position 36 with a ΔG of −8.40, −8.90. AcCoV-Duck and AcCoV-Turkey changed from U to A in the last position in sequence with ΔG of −10.20, −10.10. And deltaCoV-HKU15 and deltaCoV-HKU17 present a change from C to U in the third position with ΔG of −8.30. Likewise, it can be seen that the SARS-CoV-Tor2 variant (ΔG: −6.60 kcal/mol) shares a similar structure, probably functional with AcCoV-Duck, AcCoV-Turkey, deltaCoV-HKU15, and -HKU17, with the formation of a loop of 11rb, two loops of 7rb, and one of 6rb and one stem of 8rb or 6rb (Figure 6 and Figure S1B, Tables S5 and S12). Interestingly, the SARS-CoV, SARS-CoV-2, and SARS-CoV-Tor2 variants adopted differential structures but with probable functionality, which suggests flexibility in this *cis* element of viral genetics. These results point to the importance of s2m in the viral biology of coronaviruses and underline its role as a key functional structure in the regulation of gene expression and other viral processes.

The presence and adoption of a functional structure of the *cis* elements (stem-loop, pseudoknot stem-loop, s2m) in the alpha-, beta-, gamma-, and deltacoronavirus genera point to their relevance in the adaptation and pathogenicity of coronaviruses in different contexts and hosts. Likewise, the conformational flexibility of the s2m element in the SARS variants suggests a coevolution of this element in this viral group. For its part, the variability in secondary structures suggests genomic adaptations that may be related to replication processes, genetic regulation, as well as the specific pathogenicity of each variant.

**Figure 7:**
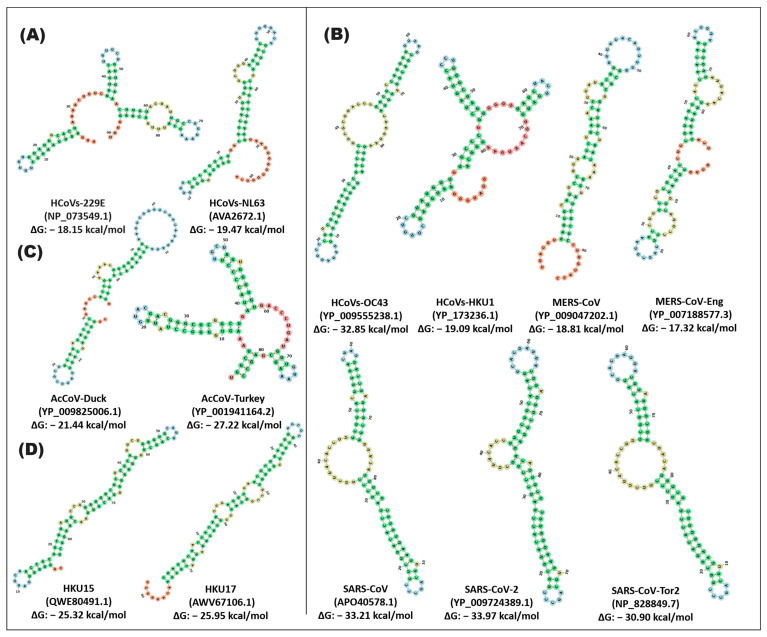
In silico secondary structure of the stem-loop1,2 unit of alpha-, beta-, gamma-, and deltacoronavirus. The prediction of the secondary structure was carried out by the Web Server RNAfold of the ViennaRNA. (**A**) alphacoronavirus; (**B**) betacoronavirus; (**C**) gammacoronavirus; (**D**) deltacoronavirus. In the secondary structures, the stem is represented by green, and the loop is represented by different colors of blue, orange, and red. In the highlighted panel, the types of coronaviruses close to SARS-CoV-2, which cause COVID-19, are grouped.

**Figure 8:**
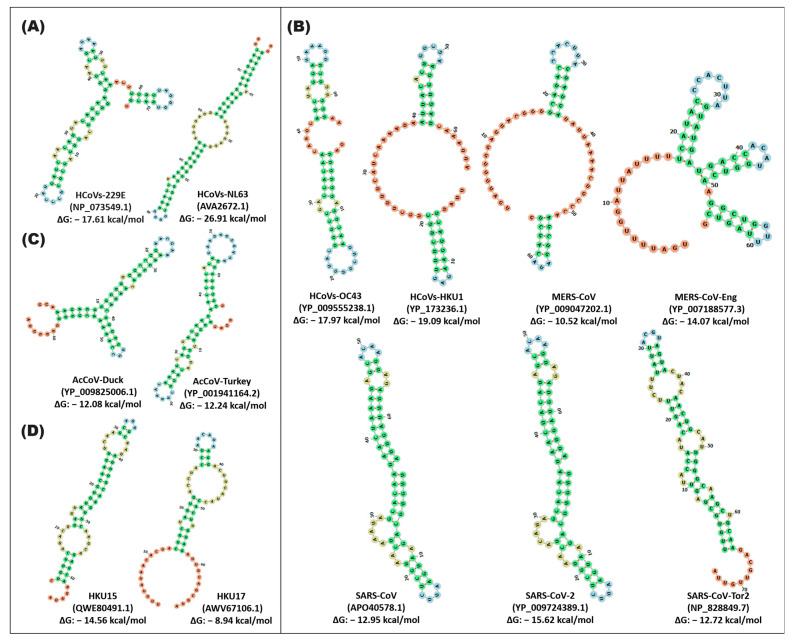
In silico secondary structure of the pseudoknot stem-loop1,2 unit of alpha-, beta-, gamma-, and deltacoronavirus. The prediction of the secondary structure of s2m was carried out by the Web Server RNAfold of the ViennaRNA. (**A**) alphacoronavirus; (**B**) betacoronavirus; (**C**) gammacoronavirus; (**D**) deltacoronavirus. In the secondary structures, the stem is represented by green, and the loop is represented by different colors of blue, orange, and red. In the highlighted panel, the types of coronaviruses close to SARS-CoV-2, which cause COVID-19, are grouped.

The comparative analysis of RNA sequences of the stem-loop1 and -2 units of the coronavirus genera indicated flexibility in the conformation of the secondary structures when they are modeled together. The SARS and DeltaCoV variants that presented a similar structure are distinguished; SARS-CoV, SARS-CoV-2, and SARS-CoV-Tor differ in four sequence changes (position 53: U-A, 56: U-C, 65:C-U, 74: U-A, -C) with a ΔG of −33.21, −33.97, and −30.90; likewise, the formation of a small loop of 7rb (position 12–18) and one stem of 18rb (position 1–9, 20–28) is observed. SARS-CoV-Tor2 shares a 21rb loop (position 33–79) and an 11rb loop (position 54–64) with SARS-CoV and SARS-CoV-2, respectively (Figure 7). For its part, in the stem-loop1 and -2 pseudoknot unit, the only observation was that the SARS-CoV and SARS-CoV-2 variants exhibited three changes, which were in position 27: A-C; 43: A-C; and 59: U-C—along with the formation of five loops (one of 9rb, two of 6rb and two of 7rb) and a stem of 26rb with a ΔG of −12.95, −15.62 (Figure 8).

The functionality of the structure of the stem-loop units 1 and 2 and pseudoknot stem-loop1-2 indicated to be functional in the coronavirus genera analyzed (Tables S6 and S7). These results suggest that the clustering of stem-loop1 and -2 elements is very important and likely plays a critical role in the viral biology of these coronaviruses. Importantly, when these *cis* elements are analyzed individually, their functionality appears to decrease. This could indicate that these elements interact in coordination to carry out specific coordinated functions and mechanisms in the viral genome. Functional analyses suggest that most *cis* elements in coronaviruses tend to adopt a structure that fits a pattern called “H”, implying that they interact in parallel and in synchrony to carry out their specific functions in the genetic regulation or other viral processes.

Furthermore, a loss of functionality of stem-loop1 and -2 was observed in deltacoronavirus, the most phylogenetically distant coronavirus variants included in this study (Figure S1). This could indicate that in the deltacoronavirus, these *cis* elements have evolved differently or that their functions have changed compared to other coronavirus genera.

Interestingly, analyzing the structure of the s2m *cis* element allows us to discriminate and group each variant within its phylogenetic genus (Figure S1). This may indicate that these s2m regions are highly adapted to specific SARS variants and may play an important role in regulating viral replication and pathogenicity in these variants. In summary, this is an important indication of the complexity of *cis* structures in coronaviruses and their ability to adapt and evolve based on viral variants. The coordination between *cis* elements and their functional importance in viral biology indicates the need for further research to fully understand their role in genetic regulation and viral processes.

### 3.3. Cis Elements in the SARS-CoV Lineage

With the premise of knowing the structural behavior of the *cis* elements according to their organization in the genome, an in silico analysis of the ORF1ab, ORF1a, and 3′-UTR of each viral variant was carried out. The stem-loop (-1, -2), pseudoknot stem-loop, and s2m are located in the ORF1ab (organization of non-structural proteins Nsp), ORF1a (ORF of structural proteins and viral accessory factors), and the extreme 3′-UTR of viral variants.

**Figure 9:**
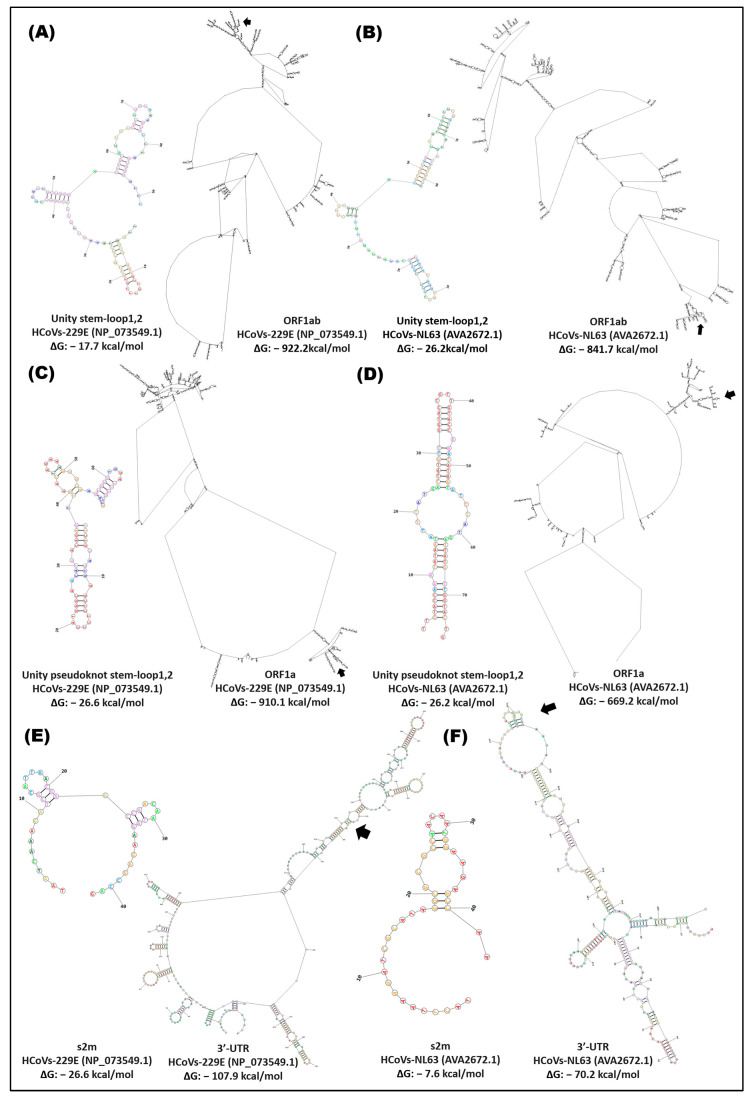
In silico secondary structure of *cis* elements and location in the ORF1ab, ORF1a, and 3′-UTR of alphacoronavirus. (**A**) HCoVs-229E, stem-loop1,2 unit, and partial ORF1ab; (**B**) HCoVs-NL63, stem-loop1,2 unit, and partial ORF1ab; (**C**) HCoVs-229E, stem-loop1,2 pseudoknot unit, and partial ORF1a; (**D**) HCoVs-NL63, stem-loop1,2 pseudoknot unit, and partial ORF1a; (**E**) HCoVs-229E, s2m, and 3′-UTR; (**F**) HCoVs-NL63, s2m and 3′-UTR. Secondary structure prediction was performed in the RNA structure server program; ~2690rb ORF1ab, ~2500rb ORF1a, and the complete 3′-UTR were analyzed.

**Figure 10:**
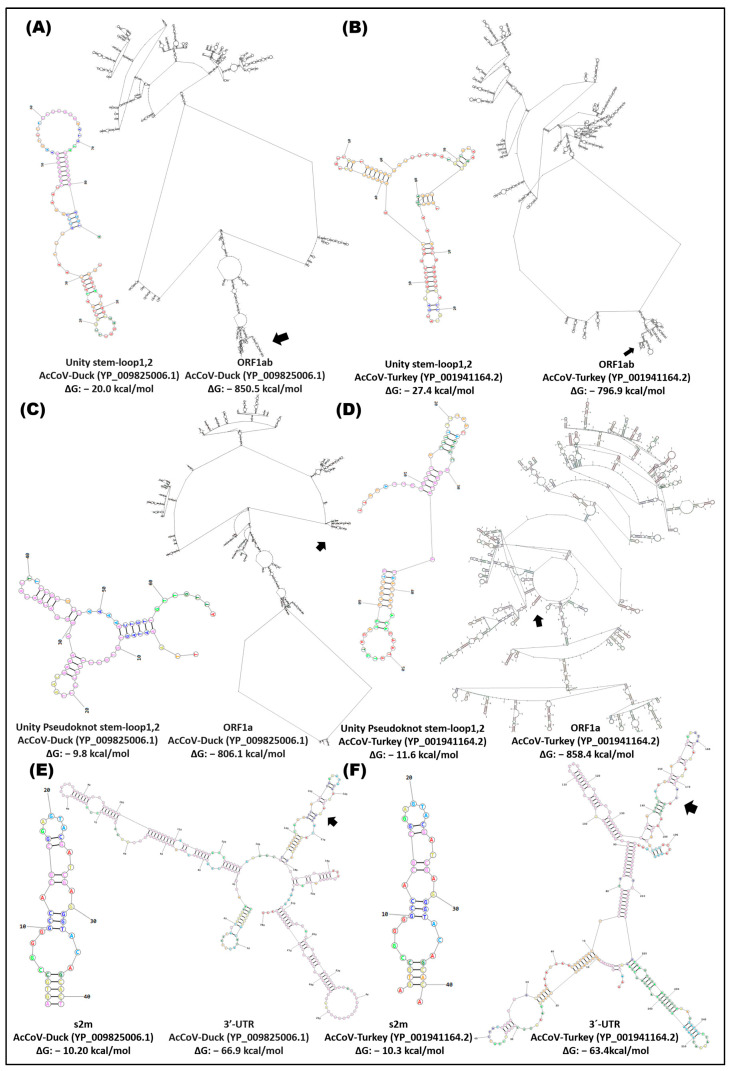
In silico secondary structure of *cis* elements and location in the ORF1ab, ORF1a, and 3′-UTR of gammacoronavirus. (**A**) AcCoV-Duck, stem-loop1,2 unit, and partial ORF1ab; (**B**) AcCoV-Turkey, stem-loop1,2 unit, and partial ORF1ab; (**C**) AcCoV-Duck, stem-loop1,2 pseudoknot unit, and partial ORF1a; (**D**) AcCoV-Turkey, stem-loop1,2 pseudoknot unit, and partial ORF1a; (**E**) AcCoV-Duck, s2m, and 3′-UTR; (**F**) AcCoV-Turkey, s2m, and 3′-UTR. Secondary structure prediction was performed in the RNA structure server program; ~2690rb ORF1ab, ~2500rb ORF1a, and the complete 3′-UTR were analyzed.

**Figure 11:**
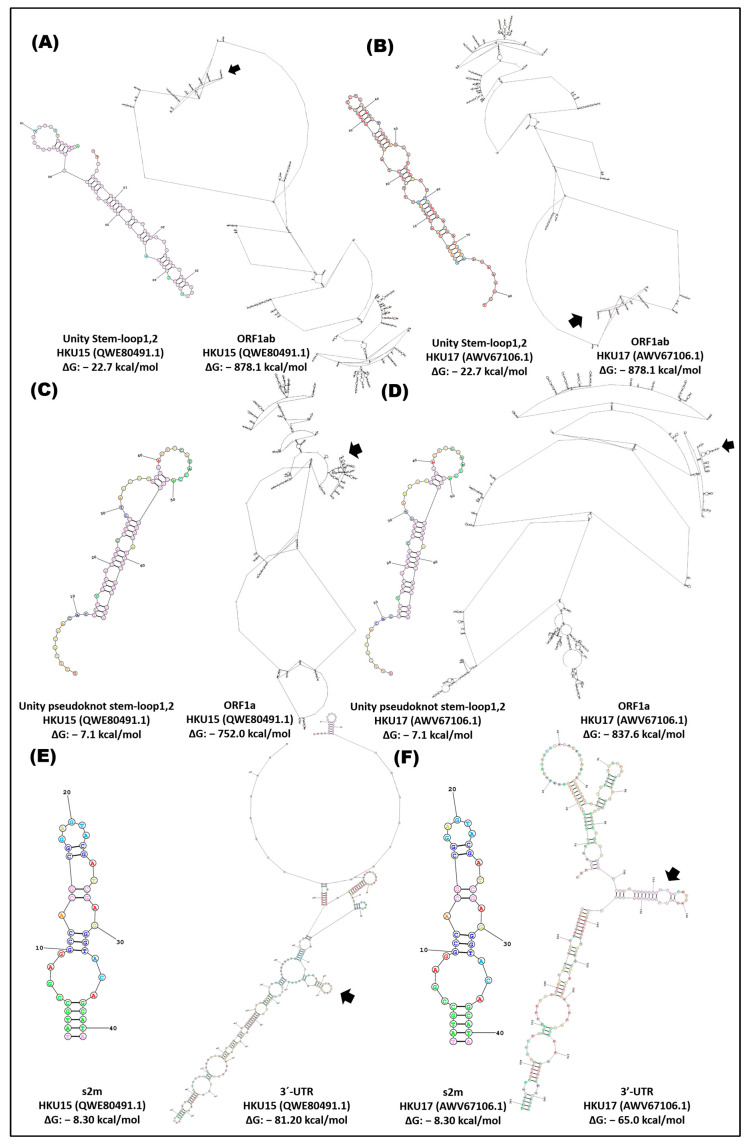
In silico secondary structure of *cis* elements and location in the ORF1ab, ORF1a, and 3′-UTR of deltacoronavirus. (**A**) CoV-HKU15, stem-loop1,2 unit, and partial ORF1ab; (**B**) CoV-HKU17, stem-loop1,2 unit, and partial ORF1ab; (**C**) CoV-HKU15, stem-loop1,2 pseudoknot unit, and partial ORF1a; (**D**) CoV-HKU17, stem-loop1,2 pseudoknot unit, and partial ORF1a; (**E**) CoV-HKU15, s2m, and 3′-UTR; (**F**) CoV-HKU17, s2m, and 3′-UTR. Secondary structure prediction was performed in the RNA structure server program; ~2690rb ORF1ab, ~2500rb ORF1a, and the complete 3′-UTR were analyzed.

The *cis* elements (stem-loop unit, stem-loop pseudoknot unit, and s2m) in the ORFs and the end 3-UTR of the alpha-, gamma-, and deltacoronavirus variants presented structural flexibility of these groups; the HCoVs-229E and HCoVs-NL63 variants had the lowest ΔG in the ORF1ab (−922.2 kcal/mol) and ORF1a (−910.1 kcal/mol), where the stem-loop1,2 unit and pseudoknot stem unit are located (stem-loop1 and -2) (Figure 9, Figure 10 and Figure 11). The 3′-UTR of the gammacoronavirus variants presented a ΔG of approximately −63.0 kcal/mol, and the s2m was −10.20 with the formation of three loops (Figure 10). Deltacoronaviruses (CoV-HKU15, CoV-HKU17) exhibited a ΔG of −81.20 and −65.0 kcal/mol with a s2m −8.30 kcal/mol (Figure 11).

**Figure 12:**
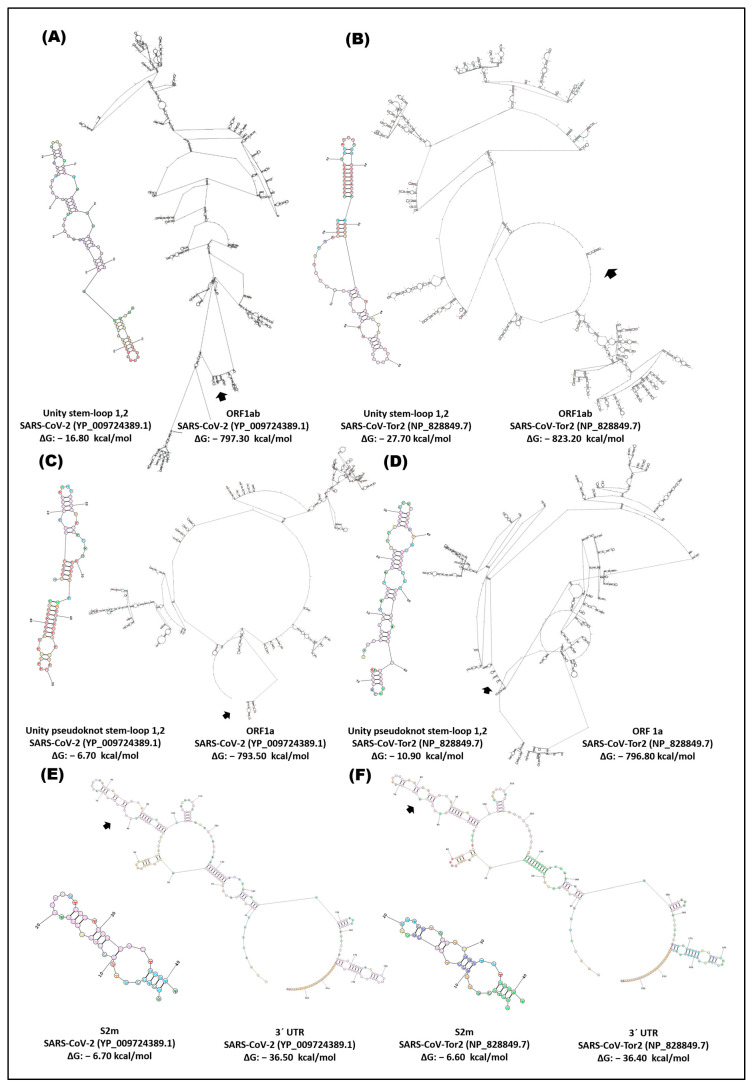
In silico secondary structure of *cis* elements and location in the ORF1ab, ORF1a, and 3′-UTR of betacoronavirus. (**A**) SARS-CoV-2, stem-loop1,2 unit, and partial ORF1ab; (**B**) SARS-CoV-Tor2, stem-loop1,2 unit, and partial ORF1ab; (**C**) SARS-CoV-2, stem-loop1,2 pseudoknot unit, and partial ORF1a; (**D**) SARS-CoV-Tor2, stem-loop1,2 pseudoknot unit, and partial ORF1a; (**E**) SARS-CoV-2, s2m, and 3′-UTR; (**F**) SARS-CoV-Tor2, s2m, and 3′-UTR. Secondary structure prediction was performed in the RNA structure server program; ~2690rb ORF1ab, ~2500rb ORF1a, and the complete 3′-UTR were analyzed.

The RNA structure of ORF1ab demonstrated a notable difference in the configuration of the stem-loop1,2 unit between the genome of SARS-CoV-2 and SARS-CoV-Tor2 (Figure 12A,B). The stem-loop is located at a distance of 3000rb downstream from the start of ORF1ab and is located within the ORF encoding the SARS non-structural proteins (Nsp), as shown in Figure 1. However, the unit pseudoknot stem-loop1,2 exhibits a similar structural pattern to stem-loop1,2; this *cis* element is located 3000 bp downstream from the start of ORF1a (Figure 12C,D). ORF1ab (ΔG: −797.30, −823.20 kcal/mol) and ORF1a (−793.50, −796.80 kcal/mol) present a differential configuration of SARS-CoV-2 and SARS-CoV-Tor2 (Figure 12).

The results obtained by analyzing the structure of the RNA in the ORF1ab of SARS-CoV-2 and SARS-CoV-Tor2 reveal an interesting disparity in the configuration of the stem-loop1,2 unit and pseudoknot stem-loop1,2 between these two viral variants (Figure 12A–D). These findings underline the complexity and diversity of RNA structures in coronavirus genomes. The variability in the configuration of these *cis* elements may be related to the adaptation of the virus to different environmental conditions or to the interaction with the host. Comparison of secondary structures in the 3′-UTR end of SARS-CoV-2 and SARS-CoV-Tor2 revealed a similarity in the conformation of s2m and the 3′-UTR end with a ΔG of −36 kcal/mol (Figure 12E,F). At the 3′ UTR end of betacoronaviruses, we identified the presence of the s2m *cis* element, with a ΔG of −6.60 kcal/mol.

In general, it was identified that the s2m of gamma, beta, and deltacoronavirus presents a structural similarity with the formation of three loops, although, in the AcCoV-Duck, AcCoV-Turkey, CoV-HKU15, and CoV-HKU17 variants, the in silico structural conformation was different (Figure 10, Figure 11 and Figure 12). It can be inferred how this segment at the 3′ end of the viral genome allows the formation of a stem-loop structure conserved in coronaviruses, which possibly plays a fundamental role in protecting this end. This suggests that the coevolution and function of s2m in different coronavirus variants could represent a genomic adaptation of betacoronaviruses such as SARS-CoV-2. This adaptation appears to be crucial for maintaining the integrity of the viral genetic material, which could target viral replication processes and increase pathogenicity. These findings emphasize the importance of understanding the conservation and function of s2m in new SARS variants and its influence on their biology and interaction with the host.

Our analysis has revealed the presence of *cis* elements in the genome of all four coronavirus genera. However, in betacoronaviruses of recent origin, conservation of s2m is observed in their structure. In these variants, these *cis* elements are located in specific regions of the genome, at 13,476–13,503 and 13,488–13,542 in SARS-CoV-2 and 13,406–13,435 and 13,418–13,472 in SARS-CoV-Tor2, and present a junction between these segments. Similarly, pseudoknot stem-loop1 and -2 are found at defined locations, at 29,609–29,644 and 29,629–29,657 in the SARS-CoV-2 genome and at 23,459–23,499 and 24,262–24,294 in SARS-CoV-Tor2 (Figure 1). This study offers us an alternative to using s2m as a potential phylogenetic marker to catalog SARS variants, although a series of analyses are missing to elucidate this objective.

In the literature, it has been described that the flexibility in the secondary structures of RNA seems to be related to critical processes of control and regulation of replication, viral pathogenesis, and changes in the ribosomal structure of the coronavirus genome. The presence and specific location of these *cis* elements in viral variants suggest their importance in the adaptation and function of these viruses in different contexts and could provide potential targets for future research and therapeutic strategies.

Ultimately, these results emphasize the continued need to investigate and understand the structure and function of *cis* elements in viral genomes, as this not only contributes to our understanding of viral biology (structure and classification of viruses) but it may also offer new perspectives for the development of therapies, more effective treatments, and prevention strategies against human pathogenic coronaviruses.

### 3.4. Evolutionary Relationships of Cis Elements of Coronavirus Pathogens of Humans

**Figure 13:**
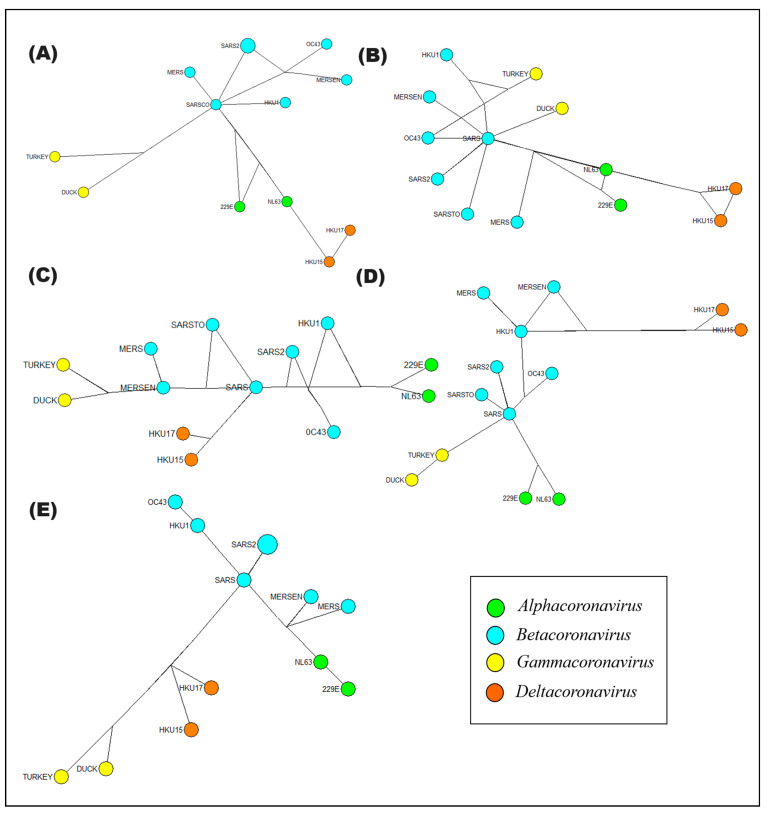
Mutational distance of *cis* elements of alpha-, beta-, gamma-, and deltacoronavirus. Variants of (**A**) stem-loop1, (**B**) stem-loop2, (**C**) pseudoknot stem-loop1, (**D**) pseudoknot stem-loop2, and (**E**) s2m. The accession numbers of alphacoronavirus: HCoVs-229E (NP_002645) and HCoVs-NL63 (NC_005831); betacoronavirus: HCoVs-OC43 (NC_006213), HCoVs-HKU1 (NC_006577), MERS-CoV (NC_19843), MERS-CoV-Eng (NC_038294), SARS-CoV (KY352407), SARS-CoV-2 (NC_04718), and SARS-CoV-Tor2 (NP_828849); gammacoronavirus: AcCoV-Duck (NC_048214) and AcCoV-Turkey (NC_010800); (**D**) deltacoronavirus: porcine coronavirus HKU15 (MW685622) and sparrow deltacoronavirus HKU17 (MG812375). The color of the circles represents the variants of *cis* elements of different taxonomic groups of coronaviruses. The line joining the variants represents the mutational distance. The minimum haplotype network was performed using the median-joining method with Network v.4.6 software.

The similarity analysis of *cis* elements initially indicated a significant separation between the phylogenetic groups of coronaviruses based on the presence and structure of specific *cis* elements (stem-loop1 and -2; pseudoknot stem-loop1 and -2; s2m), as shown in Figure S2. However, by building a haplotype network, it was revealed that variants of the SARS-CoV virus share a direct connection with the avian infectious bronchitis virus (AcCoV-Duck and AcCov-Turkey) coronaviruses. This finding suggests that betacoronaviruses, despite their genetic diversity, represent the most recent group in terms of mutational distance (Figure 13). The evolutionary relationships analyzed indicated a remarkable genetic diversity in the structure of the *cis* elements. Nevertheless, a point of convergence was observed between the alpha-, beta-, gamma-, and deltacoronavirus clusters. Despite this diversity, the haplotypes of these *cis* elements were mainly grouped within the category of betacoronaviruses. This trend surprisingly reflects what is observed in the variants within each phylogenetic group. Taken together, these results suggest that, although there is genetic diversity in the structure of *cis* elements among different coronavirus groups, betacoronaviruses share significant evolutionary similarities. This raises intriguing questions about the role of the structure of *cis* elements in the adaptation and genetic regulation of these viruses in several contexts.

These results accentuate the complexity of the evolutionary relationships between coronaviruses and draw attention to the importance of further investigating the role of *cis* elements and their presence in the viral genome. This research offers valuable information from using these *cis* elements to trace the lineage of different groups of coronaviruses to the possibility of using s2m to classify the new SARS-CoV variants currently influencing the population and to fully understand pathogenicity and adaptation of coronaviruses.

## 4. Discussion

### 4.1. Importance of Cis Elements in Human Pathogenic Coronaviruses

The identification of the organization of *cis* elements in human pathogenic coronaviruses and their genetic mapping has been a fundamental area of research for understanding the biology and pathogenicity of these viruses. In this context, it is important to highlight that *cis* elements are regulatory sequences that play a crucial role in the regulation of gene expression and viral replication. As different variants of the coronavirus genome have been studied, significant variation in the position, location, and length of these *cis* elements has been observed, which may have important implications on virulence and viral replication capacity.

One of the main conclusions that emerge from this research is that the organization of *cis* elements in coronaviruses is not static but diverges according to the viral variant. This variability can be due to several reasons, including genetic mutations that occur over time due to the rate of evolution of these viruses. These mutations can affect the sequences of *cis* elements and thus influence their function and location in the viral genome.

The disparity in the organization of *cis* elements in different coronavirus variants can have diverse significant implications, such as the regulation of gene expression host immune response, and be useful in the development of antiviral treatments and vaccines. Changes in the location or length of these *cis* elements can influence the expression of key viral proteins, which can affect the virulence and ability of the virus to replicate. This variability also can influence the interaction between the virus and the host’s immune system.

These changes can result in the evasion of the immune response or the generation of new adaptive immune responses. The variability in these elements may require therapeutic strategies adapted to the specific genetic characteristics of each variant.

In summary, the identification of the organization of *cis* elements in human pathogenic coronaviruses and the observation of variations in their position, location, and length in different viral variants highlight the complexity of viral biology. In addition, it highlights the importance of continuing to advance in the development of genetic markers that allow us to know the phylogenetic and lineage relationships to understand the evolution of these coronavirus variants and to be prepared for the emergence of a new viral variant and an eminent pandemic. These findings have important implications for understanding viral pathogenicity, designing therapeutic strategies, and the host immune response to these highly relevant pathogens.

### 4.2. Coevolution of Cis Elements in the Genome of Human Pathogenic Coronaviruses

SARS-CoV-2, responsible for the COVID-19 pandemic, possesses a positive-stranded RNA genome with a length of approximately 29,800 nucleotides. This genome is organized into two open reading frames (ORFs): ORF1ab and ORF1a. ORF1ab codes for a number of non-structural proteins (Nsp1 to Nsp16), which play essential roles in proteolytic processing of polyprotein 1ab, viral genome replication, and synthesis of ARN. Polyprotein 1ab is the translation product of ORF 1a and 1ab and is generated by a ribosomal shift mediated by the pseudoknot stem-loop1,2 [21]. ORF1a codes primarily for structural proteins that play a critical role in virion formation, including structural spike protein (S), envelope protein (E), membrane glycoprotein (M), and nucleocapsid protein (N). These viral components are essential for the structure and function of the virus. At the terminal ends of the viral genome, there are two untranslated regions: the 5′-UTR (untranslated region at the 5′ end) and the 3′-UTR (untranslated region at the 3′ end). In the 5′-UTR, short ORFs encoding peptides of approximately 3 to 11 amino acids have been identified, such as the stem-loop1,2 [22]. Alternatively, the 3′-UTR a stem-loop II type motif upstream of the poly(A) 3′-terminal tail, known as s2m. These *cis* elements, including stem-loop, pseudoknot, and s2m, play a critical role in interacting with viral proteins, host proteins, and other accessory RNA factors. These interactions are crucial for regulating key processes, such as translation, viral genome replication, and virus encapsulation [23].

The adaptation of *cis* elements to the genome of viral variants, such as the stem-loop (-1, -2) and s2m, at the 5′ and 3′ terminal ends raises interesting implications for carrying out their regulation and viral cycle. In particular, stem-loop1 and -2, which are located within the ORF1ab encoding non-structural proteins (Nsp1-16), have been previously linked to the stimulation of ribosomal frameshifting. This element exhibits an RNA loop structure along with posterior regions, and it can form pseudoknot structures. It is important to note that these structures are not static, and their conformation can vary, which adds an additional layer of complexity. For example, a 32-nucleotide sequence downstream of the stem of the pseudoknot has been reported to be complementary to a part of the loop, suggesting flexibility in structure. Furthermore, it has been mentioned that another stem-loop structure can form, spanning about 150 nucleotides downstream, and that this conformation can interact with other members of the same family to form interconnected stem-loops [24]. The proper structural conformation of these elements is of particular interest due to their potential impact on the viral growth rate and the regulation of viral protein synthesis. The interaction between these structures and the ribosome can cause a change in the ribosomal framework, resulting in the production of an incomplete polypeptide. This, in turn, can have significant effects on viral replication and the virus life cycle. The conservation and structural dynamics of *cis* elements, such as stem-loop (-1, -2) and s2m, in the SARS-CoV-2 genome suggest their relevance in the fitness of the virus. These elements may play a fundamental role in the regulation of viral protein synthesis and its replication strategy. Detailed study of these structures provides a deeper understanding of the mechanisms of SARS-CoV-2 and may have important implications for the development of therapeutic strategies aimed at interrupting its replication cycle.

The frameshift phenomenon in coronaviruses was initially documented in avian infectious bronchitis virus (IBV) and has since been observed in other members of the coronavirus family. This process is essential for the expression of certain viral genes, and it is important in the replication and synthesis of key components of the virus. In the case of IBV, it has been identified that a specific fraction of the ribosomes that are involved in the translation of ORF1a changes the reading frame in a precise place. This allows these ribosomes to decode the information contained in ORF1b. This precise translation is essential for the synthesis of viral RNA-dependent RNA polymerase and other components necessary for viral replication. ORF1b lacks a separate site for translation initiation, further highlighting the importance of this frame-shifting mechanism. This frame-changing process provides an efficient and highly regulated strategy for the expression of specific viral genes, ensuring that the ORF1b product is expressed at precise levels relative to the ORF1a product. This regulation is critical to the virus cycle and its ability to replicate and spread effectively. Previous studies have described that the mechanism of frame change in coronaviruses is regulated by *cis* elements. This knowledge is fundamental for the development of therapeutic and research strategies aimed at combating coronavirus infections, including the COVID-19 pandemic [25].

The conservation of the stem-loop motif II (s2m) element in various families of positive-sense single-stranded RNA viruses is an intriguing finding that showcases the potential importance of this motif in the viral cycle and its interaction with their hosts. In the literature, s2m has been shown to be conserved not only among Coronaviridae viruses but also within the distantly related families Astroviridae, Caliciviridae, and Picornaviridae [26,27]. This study focuses on the identification of structural and functional conservation of s2m in SARS variants and other members of the coronavirus lineage in the final 3′-UTR region, specifically within the terminal hypervariable portion (HVR). Although the exact function of the s2m element has not yet been elucidated, its presence in multiple virus families suggests a fundamental role in the replication and persistence strategy of these pathogens. Several functions have been proposed for s2m, such as the sequestration of host protein synthesis, its participation in RNA interference pathways, and its contribution to RNA recombination events [28]. These potential functions highlight the versatility of s2m and its ability to influence multiple aspects of virus–host interaction. The broad conservation of s2m in different families of single-stranded RNA viruses suggests that this motif may play a crucial role in the adaptation and survival of viruses in various environments. In addition, understanding the coevolution and function of s2m could have important implications in virus detection and the development of therapeutic strategies. The ability to identify an element shared by diverse virus families could open new avenues for the development of broad-spectrum antivirals and vaccines. The presence of the s2m element in distant virus families underlines its importance in viral function and accentuates its potential as a therapeutic target [29]. As research in this area continues, it is likely that more will be revealed about the precise functions of s2m and its relevance in the fight against various viral diseases.

The 3′-UTR region of coronaviruses is a critical part of their genome that contains a conserved pseudoknot structure, playing an essential role in viral genome replication [30]. However, it has been observed that this region exhibits variability in its structure between different coronaviruses, as documented in the viruses MHV, BCoV, and HCoV-229E, which varies from the formation of the fork to the conservation of an extremely large stem-loop and a pseudoknot (PK) [31]. For example, it has been shown that in BCoV and MHV, the conformation of a fork-like PK is crucial for the binding of canonical RdRp, underscoring its importance in viral replication [32,33]. An interesting discovery is the overlapping of BSL (bulge stem-loop) and PK regions in BCoV and MHV, suggesting that these two structures cannot coexist simultaneously. This raises the possibility that these elements may adopt substructures that function as a “molecular switch” capable of controlling the transition between different stages of the viral replication cycle [34]. This structural dynamic could be essential to regulate viral replication and ensure that transcription and replication events occur at the right time and in the right sequence. The results of this study suggest that the thermodynamic conformations acquired by the stem-loop1-2, pseudoknot stem-loop1 and -2, and mostly s2m elements in the different coronavirus genera are characteristic of each variant. This structural flexibility is consistent with the idea that these regions at the 5′-UTR terminal end can function as a molecular switch. This could be critical for specific interactions with transcription factors, which in turn could trigger activation or repression of viral replication. The variability in the structure of the 3′-UTR region of coronaviruses and the possibility that it acts as a molecular switch underscore the complexity of viral regulation to complete the life cycle processes of coronaviruses. This could be critical for specific interactions with transcription factors and protein interaction, which in turn could trigger activation or repression of viral replication. Nonetheless, the role of each cis element in coronaviruses in activating genes necessary for viral replication, transcription of structural proteins, and modulation of the host immune response is so far unknown, which is why it is important to direct scientific research toward this field.

In this study, the analysis of the RNA structures of the genetic unit of stem-loop (-1, -2) and pseudoknot stem-loop (-1, -2) in various genera of coronavirus reveals remarkable structural functionality in these *cis* elements. These results highlight the importance of maintaining these elements in the genome of coronaviruses, and they appear to be functional. It is particularly interesting to note that these *cis* elements show increased functionality when acting together, suggesting essential coordination between them to perform specific functions in the viral genome. These results suggest that these elements act as essential genetic units in gene regulation and other viral processes in a wide range of coronaviruses. Overall, these results highlight the critical importance of *cis* elements in coronavirus activity and underscore the need to better understand their function and regulation. This evidence has important implications for the investigation of *cis* elements and understanding how they have been maintained during the coronavirus lineage until the emergence of the new variants of SARS-CoV-2.

### 4.3. Functional Adaptation of Cis Elements in Coronaviruses

The study of the conformation and functionality of the *cis* elements in the betacoronavirus offers a vision of the evolution of SARS-CoV-2 variants and their relationship with ancestral lineages of coronaviruses, such as delta and gamma. The identification of a probably functional flexible conformation in the stem-loop1 and -2, pseudoknot, and s2m elements in coronaviruses raises important questions about the coevolution of these *cis* elements and their impact on viral pathogenicity.

It is notable that although the conformation of the 3′-UTR end in the coronavirus variants adopts a particular structure in each accession, the s2m element presents a similar structure in gamma (AcCoV-Duck, AcCoV-Turkey), delta (HKU15, HKU17), and beta (SARS-CoV-2, SARS-CoV-Tor2) and is present in the viral trajectory of the lineage. This result could indicate a selective evolutionary pressure that favors the retention of these structures in coronavirus variants, possibly due to their crucial role in virus replication and transmission. Furthermore, the presence of *cis* elements in variants from ancestral coronavirus lineages implies that these structures play a vital role in the biology of the virus, suggesting continuous adaptation over time. The suggestion of possible coevolution between *cis* elements in recent SARS-CoV-2 variants opens a new research window. This result raises the possibility that interactions between these *cis* elements are essential for efficient virus replication. A better understanding of this coevolution could provide valuable information for the development of more effective antiviral therapies and prevention strategies.

The implication of these results in the evolution of pathogenic entities for humans is of great relevance. The conservation of these *cis* elements in the viral genome highlights the importance of these components in the virus’s ability to infect and spread in human populations. This could have important implications for public health, especially in the context of the continued evolution of SARS-CoV-2 and the emergence of new variants. Our study highlights the evolutionary importance of *cis* elements in the evolution along with the genome of variants of SARS-CoV-2 and other related coronaviruses. The proposed coevolution between these *cis* elements suggests additional complexity in viral biology that deserves continued attention in future research. Our research not only contributes to fundamental scientific knowledge about coronaviruses but is also involved in the search for genetic biomarkers that allow us to know the origin and trajectory of the new coronavirus variants that currently affect the population.

Betacoronavirus is the group that marked an antecedent in viral transmission to the human host. In this regard, the question arises whether this *cis* element would allow us to follow the origin trajectory of the new variants SARS, OMICRON, ERIS, and recently PIROLA. The origin of the SARS-CoV-2 virus is proposed to be the result of homologous recombination between bat and pangolin coronaviruses, likely triggering interspecies transmission to humans [35,36]. High rates of recombination among coronaviruses have been documented, although the exact mechanism by which this occurs is unknown [37]. Similarly, this high rate of recombination has been studied extensively in other viral families, such as Astroviridae, Caliciviridae, and Picornaviridae [38]. Interestingly, all of these viral families contain the s2m element within the 3′-UTR [26,39]. In 2022, Imperatore et al. [1] proposed that given the high degree of similarity between the s2m element recombination events within these viral families, this *cis* element may be involved in genomic dimerization and potentially contribute to subsequent homologous recombination events of the coronavirus lineage. Furthermore, during the COVID-19 pandemic, many genomic sequences of isolates from Australian patients with SARS-CoV-2 have deletions or mutations in s2m, suggesting that RNA recombination may have occurred in this RNA element [40,41,42]. In this study, the identification of the secondary structure of the s2m *cis* element was observed, exhibiting differential patterns in the alpha, beta, gamma, and delta phylogenetic groups of the coronavirus, probably reinforcing the incidence of homologous recombination or acting as an RNA binding site.

In recent years, it has become increasingly evident that coronaviruses use various components of the host cell for their own replicative benefit [43]. Bioinformatic analysis of the SARS-CoV-2 genome has revealed several potential miRNA binding sites within the 5′ and 3′-UTR, including two potential binding sites within the s2m element, which leads to a sequestration of the miRNA from the host cell favoring viral stability [44,45]. In addition, in North American eastern equine encephalitis virus (EEEV), sequestration of miRNA-142-3p results in the repression of innate immune responses and subsequently increased disease severity [46]. In most circumstances, the location of natural miRNA binding sites occurs within the 3′UTRs of viral RNA genomes [47].

The role of host cell miRNA sequestration by viruses has been broadly demonstrated in previous studies, which normally benefits the viral life cycle. Another potential role of miRNA-1307-3p in the life cycle of SARS-CoV-2 relates to the mechanisms of viral entry and spread. For example, recognition of ACE2 receptors on ATII lung epithelial cells by the SARS-CoV-2 S protein is generally considered to be the first step in viral entry and subsequent infection [48]. During host invasion by viral RNA, s2m first binds to one or more proteins as a mechanism for viral RNA to replace genetic information for host protein synthesis. This molecular manipulation strategy highlights the sophistication of the viral machinery and the importance of the s2m element in the evolution strategy of the virus. The element s2m has been identified as an effective target for the design of antiviral drugs and antisense oligonucleotides [49].

The s2m *cis* element is a critical genetic component that has been identified in coronavirus variants and has been associated with their virulence and ability to spread. Understanding how this element affects viral replication and the host immune response is essential to develop effective therapeutic strategies [50]. This study provides strong evidence for the importance of *cis* elements in the virus life cycle and highlights the need for additional research to explore their potential as therapeutic targets.

One of the most important implications of this study is its contribution to the development of tools to mitigate severe symptoms of COVID-19. By understanding how *cis* elements affect virus virulence and spread, researchers can design specific therapies that target these elements to disrupt their key functions. This could include the development of drugs that block the action of the stem-loop1 and -2 and s2m elements or modulate their activity to reduce the virus’s ability to cause severe disease.

This study also offers insights into the design of therapies that can slow viral spread. By better understanding the mechanisms by which *cis* elements affect viral replication and entry into host cells, therapeutic strategies that interfere with these processes can be developed [50]. This could include the design of specific viral inhibitors that block the function of the *cis* element and reduce the ability of the virus to infect new cells.

These advances are especially significant in the context of the global COVID-19 pandemic. The ability to develop specific therapies targeting key elements in the biology of the virus can make a difference in managing the disease and reducing its impact on public health. Furthermore, these findings offer opportunities for future scientific research in the field of viral diseases, providing a solid foundation to address future viral emergencies more effectively.

### 4.4. Future Research

Future research on the prediction of RNA structures based on free energy minimization may have significant implications in the scientific study of SARS-CoV-2, the virus responsible for the COVID-19 pandemic. Some areas of research in which this application intervenes are developing predictive models of the RNA structures present in the SARS-CoV-2 genome. This could help to better understand the structure and function of key elements of the virus, such as regulatory regions, protein binding sites, and regions critical for viral replication. It opens the possibility of studying the interactions between the RNA structures of SARS-CoV-2 and other molecules, such as viral proteins or cellular RNA molecules, which may provide crucial information about the molecular mechanisms involved in viral infection, virus replication, and the host immune response. It helps us analyze how mutations in the SARS-CoV-2 genome affect the predicted RNA structures to understand the evolution of the virus, the emergence of variants of concern, and their impact on transmissibility, virulence, and response to vaccines and treatments. Employing information from predicted RNA structures to design and develop therapies specifically directed against SARS-CoV-2, such as RNA inhibitors or modulators of RNA-protein interactions, may be a promising strategy to combat the pandemic and future viral threats. Investigating the stability of predicted RNA structures under different conditions, such as variations in temperature, pH, or the presence of co-factor molecules, can provide valuable information on the resistance of the virus in different environments and its ability to persist and transmit.

The prediction of RNA structures based on free energy minimization and the selection of structures with the lowest free energy are fundamental strategies in bioinformatics. The underlying premise is that structures with lower free energy are more stable and therefore more likely to be biologically relevant. However, it is important to keep in mind that nature does not always adopt the most stable structure due to the complex dynamics and specific conditions of the cellular environment. Thermodynamic stability is a crucial factor in the formation of RNA structures, but other factors, such as the presence of proteins, evolutionary selective pressure, and interactions with other cellular components, also influence the final structure adopted by the RNA. Therefore, although free energy-based structure prediction provides important insight, it is not sufficient to determine biological relevance alone. Future research in this field aims to complement these bioinformatic predictions with experiments that validate the stability and function of the predicted structure under real biological conditions. Some complementary analyses may include mutation analysis to evaluate the robustness of the structure against changes or interaction studies with proteins to understand their molecular function, among others.

The accuracy of RNA secondary structure predictions made by bioinformatics tools can vary depending on the complexity of the sequences and the inherent limitations of the algorithms used. It is important to recognize that, in many cases, secondary structure predictions may not be completely accurate, especially in highly variable regions or with complex folding. The validity of comparisons between less accurate secondary structure predictions depends on the context and purpose of the research. Secondary structure predictions are based on theoretical models and algorithms that may not fully capture the complexity of molecular interactions in all sequences. Therefore, it is common for predictions to not reach 100% accuracy. Although individual predictions may not be highly accurate, relative comparisons between different sequences or conditions may still be informative. If secondary structures of related sequences are being compared, discrepancies in individual predictions may reveal patterns of conservation or structural change indispensable for understanding critical biological functions that provide an evolutionary strategy in the adaptation of SARS-CoV-2 and other viruses. Predictions of RNA secondary structures may not be perfect; they are still valuable tools to understand the dynamics and function of RNA molecules of great biological value, such as *cis* elements.

The presence of suboptimal structures close to the most stable one suggests the existence of alternative conformational states that the RNA can assume, as observed in the modeling of the structures of the *cis* elements of the coronavirus variants. These states may be associated with specific biological functions, such as regulation of gene expression, interaction with proteins or other molecules, or participation in dynamic folding processes. Suboptimal structures reflect the structural flexibility of RNA and its ability to adopt different functional conformations, providing new insights into its function and regulation and fostering a more complete understanding of the RNA structural and functional diversity of *cis* elements in the viral genome.

## 5. Conclusions

**Figure 14:**
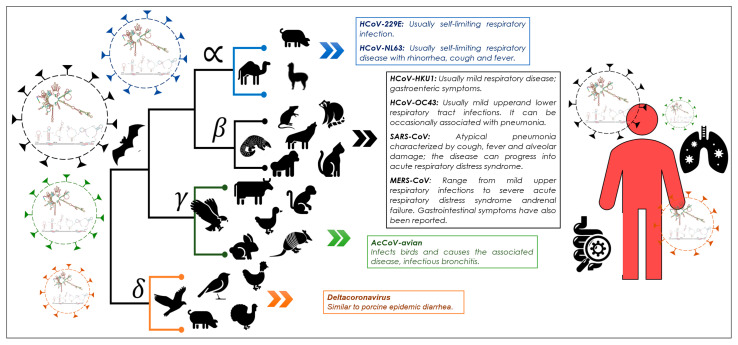
Coevolution of *cis* elements in different coronavirus genera until the origin of the betacoronavirus lineage B SARS-CoV-2 causing the COVID-19 pandemic. The *cis* elements stem-loop1 and -2, pseudoknot stem-loop1 and -2, and s2m participate in the regulation of gene expression and play a crucial role in virus adaptation and evolution. Coronaviruses are divided into four different genera (alpha, beta, gamma, and delta). Alpha-CoV and beta-CoV circulate in mammalian hosts, while gamma-CoV and delta-CoV primarily infect birds. As for SARS-CoV and MERS-CoV, molecular dynamics studies estimated that they diverged from bat CoVs in the last three decades. The first coronaviruses in humans (HCoV), HCoV-E229 and HCoV-OC43, are frequent etiological agents of seasonal acute respiratory infections, while SARS-CoV and MERS-CoV are responsible for severe acute respiratory syndromes. SARS-CoV-2 in the city of Wuhan, Hubei province in China, in December 2019, was initially epidemiologically linked to animal markets. First, SARS-CoV-2 affects the respiratory tract with atypical pneumonia and, in severe cases, causes systemic inflammation with the release of cytokines that can cause rapid deterioration, circulatory and respiratory failure, and coagulation disorders. Common symptoms of COVID-19 include fever, cough, shortness of breath and hypoxemia, muscle pain, sore throat, rhinorrhea, headache and confusion, and pneumonia, in addition to gastrointestinal problems, hypogeusia, and hyposmia. It is believed that the first SARS coronavirus originated in bats, later jumping to other small mammal species, such as the civet, and finally to humans. On the other hand, SARS-CoV-2’s intermediate host is the pangolin. All human coronaviruses have a zoonotic origin; for SARS-CoV, MERS-CoV, HCoV-NL63, and HCoV-229E, the natural reservoir is bats, and for HCoV-OC43 and HKU1, the reservoir is probably rodents. HCoV-229E presents clinical manifestations of a generally self-limited respiratory infection. Pneumonia has been reported in immunocompromised patients. Its possible origin is vector bats, and the intermediate host is possibly camels. For HCoV-NL63, clinical manifestations are generally self-limited respiratory disease with rhinorrhea, cough, and fever. It may be associated with obstructive laryngitis in children. Possible vector of origin: bats. HCoV-HKU1’s clinical feature include generally mild respiratory disease; it can occasionally cause pneumonia in infants, the elderly, and immunocompromised patients, and it is associated with gastroenteric symptoms. Possible origin and intermediate host are rodents. HCoV-OC43 patients have been detected with generally mild infections of the upper and lower respiratory tract. Occasionally, it may be associated with pneumonia. Possible origin: rodents; intermediate host: cattle. Variants derived from SARS-CoV exhibit clinical features such as atypical pneumonia characterized by cough, fever, and alveolar damage; the disease can progress to acute respiratory distress syndrome. Possible origin: bats; intermediate host: viverrids/caniformes. The global fatality rate is 10%, according to the World Health Organization (WHO). MERS-CoV betacoranavirus variants range from mild upper respiratory tract infections to severe acute respiratory distress syndrome and adrenal insufficiency. It comes from bats, and its intermediate host is camelids. The global fatality rate is 36% (WHO). The gammacoronaviruses AcCoV-Duck and AcCoV-Turkey infect birds and cause the associated disease infectious bronchitis. It is a highly infectious avian pathogen that affects the respiratory tract, intestine, kidney, and reproductive systems of chickens. Possible origin is bats, and intermediate host is birds. For deltacoronavirus, HKU15 is the causal agent of contagious gastrointestinal disease of pigs. Clinical signs include diarrhea, dehydration, vomiting, and produces atrophy of the intestinal villi. Turkey coronavirus, HKU17, is the cause of an acute and highly contagious enteric disease of turkeys; it has also been associated as a cause of enteritis in birds and mortality syndrome, characterized by high mortality, severe growth depression, and immune dysfunction. Possible origin is bats, and intermediate host is domestic pigs/birds. The study of the evolution and fixation of *cis* elements together with the virus as a genome evolution strategy of the variants of each genus of coronavirus provides valuable information on their adaptation to different environments and hosts, as well as on possible changes in their pathogenicity and transmission capacity; particularly, the SARS-CoV-2 that recently derived from the COVID-19 vaccination has demonstrated rapid genetic dynamism arising in the appearance of new variants (OMICRON, KRAKEN, ERIS, and PIROLA) with a high contagion capacity and transmission (and vice versa) from domestic animals to humans.

In recent years, various biochemical, genetic, and bioinformatics studies have provided important new knowledge about *cis*-acting elements, their relevant role in the viral replication cycle, and the strategies they adopt in host infection. This bioinformatic study suggests that the structural flexibility of cis-element RNA may be related to the adaptation strategy of the viral entity. Furthermore, this study provides evidence that the function and evolution of *cis* elements are essential for the viral life cycle of coronaviruses, as well as their replication, expression, and genome packaging. Therefore, this research suggests a coevolution of the RNA structure of the *cis* elements in the genome of viral variants together with the viral replication machinery. The presence and incidence of *cis* elements in the B lineage of coronaviruses suggest the importance of these regions in the origin and evolution in humans of these pathogenic entities. However, there are still aspects to study, such as the different interaction mechanisms that *cis* elements have adopted, the role played by the 3′ and 5′ UTR terminal regions of coronavirus variants in viral biology, and/or specific pathogenic routes between virus and host. Our results contribute to knowledge about the adaptation of *cis* elements in the viral trajectory of the B lineage in which SARS-CoV-2 stands out, in addition to its potential importance as a therapeutic target for both COVID-19 and other diseases related to the virus. More research in this area is essential to deepen our understanding of viral biology and develop more precise approaches to combat SARS-CoV-2 and other related viruses in the future. Coronaviruses are divided into four different genera (alpha, beta, gamma, and delta). The study of possible genetic markers, such as *cis* elements and structural proteins, contributes to understanding the taxonomy and trajectory of the host–vector of these viruses. This is essential to develop strategies in the field of health sciences that help mitigate new pandemics (Figure 14).

## Figures and Tables

**Table 1:** Genomes of coronavirus taxonomic genera.

Type of Coronavirus	Denomination	Host	Accession of Genome	1ab Polyprotein (ORF1ab)	Length of Genome (nt)
Alphacoronavirus					
HCoVs-229E	Human coronavirus 229E	Human, vertebrate strain: 229E	NC_002645	NP_073549.1	27,317
HCoVs-NL63	Human coronavirus NL63	Human strain: Amsterdam I	NC_005831	AVA2672.1	27,553
Betacoronavirus					
HCoVs-OC43	Human coronavirus OC43	Human, vertebrates strain: ATCC VR-759; serotype: OC43	NC_006213	YP_009555238.1	30,741
HCoVs-HKU1	Human coronavirus HKU1	Human isolate: HKU1	NC_006577	YP_173236.1	29,926
MERS-CoV	Middle East respiratory syndrome-related coronavirus	Human, vertebrate strain: HCoV-EMC; isolate: HCoV-EMC/2012	NC_19843	YP_009047202.1	30,119
MERS-CoV-Eng	Middle East respiratory syndrome-related coronavirus, England	Human, vertebrate strain: England 1; isolate: H123990006	NC_038294	YP_007188577.3	30,111
SARS-CoV	Severe acute respiratory syndrome-related coronavirus	Human, vertebrate Strain: BtKY72	KY352407 ^a^	APO40578.1	29,274
SARS-CoV-2	Severe acute respiratory syndrome-related coronavirus 2	Human, vertebrate isolate: Wuhan-Hu-1	NC_045512	YP_009724389.1	29,903
SARS-CoV-Tor2	Severe acute respiratory syndrome-related coronavirus Tor2	Human isolate: patient #2 with severe acute respiratory syndrome (SARS)	NC_04718	NP_828849.7	29,751
Gammacoronavirus ^b^					
AcCoV-Duck	Duck avian coronavirus	Human, vertebrate isolate: DK/GD/27/2014	NC_048214	YP_009825006.1	27,754
AcCoV-Turkey	Turkey avian coronavirus	Human, vertebrate isolate: MG10	NC_010800	YP_001941164.2	27,657
Deltacoronavirus ^c^					
Porcine coronavirus	Porcine coronavirus HKU15	Porcine isolate: PDCoV/Haiti/Human/0081-4/2014	MW685622	QWE80491.1	25,444
Sparrow deltacoronavirus	Sparrow deltacoronavirus HKU17	Passeridae isolate PDCoV/Haiti/Human/0081-4/2014	MG812375	AWV67106.1	25,795

^a^ Complete genomic sequence of BtKY72, which is closely related to BtCoV/BM48-31/Bulgaria/2008, a severe acute respiratory syndrome (SARS)-related virus from Rhinolophus bats in Europe. ^b,c^ Group of coronaviruses with a route of transmission through birds and porcine.

## Data Availability

Data is contained within the article and Supplementary Materials.

## Supplementary Materials

The following supporting information can be downloaded at https://www.mdpi.com/article/10.3390/cimb46060344/s1. Figure S1: Sequence alignment of *cis* elements in alpha-, beta-, gamma-, and deltacoronavirus. (A) stem-loop1; (B) s2m; (C) pseudoknot stem-loop1; (D) pseudoknot stem-loop2; (E) stem-loop2. Alphacoronavirus: HCoVs-229E (NP_073549.1), HCoVs-NL63 (AVA2672.1). Betacoronavirus: HCoVs-OC43 (YP_009555238.1), HCoVs-HKU1 (YP_173236.1), MERS-CoV (YP_009047202.1), MERS-CoV-Eng (YP_007188577.3), SARS-CoV (APO40578.1), SARS -CoV-2 (YP_009724389.1), SARS-CoV-Tor2 (NP_828849.7). Gammacoronavirus: AcCoV-Duck (YP_009825006.1), AcCoV-Turkey (YP_001941164.2). Deltacoronavirus: Porcine coronavirus HKU15 (QWE80491.1), sparrow deltacoronavirus HKU17 (AWV67106.1). Figure S2: Evolutionary relationships of *cis* elements in human pathogenic coronaviruses. Table S1: Evolutionarily conserved RNA secondary structures of stem-lopp1 of coronavirus. Table S2: Evolutionarily conserved RNA secondary structures of stem-lopp2 of coronavirus. Table S3: Evolutionarily conserved RNA secondary structures of pseudoknot stem-lopp1 of coronavirus. Table S4: Evolutionarily conserved RNA secondary structures of pseudoknot stem-lopp2 of coronavirus. Table S5: Evolutionarily conserved RNA secondary structures of s2m of coronavirus. Table S6: Evolutionarily conserved RNA secondary structures of stem-loop unit of coronavirus. Table S7: Evolutionarily conserved RNA secondary structures of pseudoknot stem-loop unit of coronavirus. Table S8: Changes in stem-loop1 ribonucleotides of coronavirus variants. Table S9: Changes in stem-loop2 ribonucleotides of coronavirus variants. Table S10: Changes in pseudoknot stem-loop1 ribonucleotides of coronavirus variants. Table S11: Changes in pseudoknot stem-loop2 ribonucleotides of coronavirus variants. Table S12: Changes in s2m ribonucleotides of coronavirus variants.
