# Supplementary material for: Genomic Evolution Strategy in SARS-CoV-2 Lineage B: Coevolution of Cis Elements
Source: Curr Issues Mol Biol. 2024 Jun 9;46(6):5744–76. doi: 10.3390/cimb46060344 (PMC11203041; doi:10.3390/cimb46060344)
Supplement: Supplementary file 1 [file cimb-46-00344-s001.zip › cimb-2983003-supplementary.pdf]

SUPPLEMENTARY MATERIAL

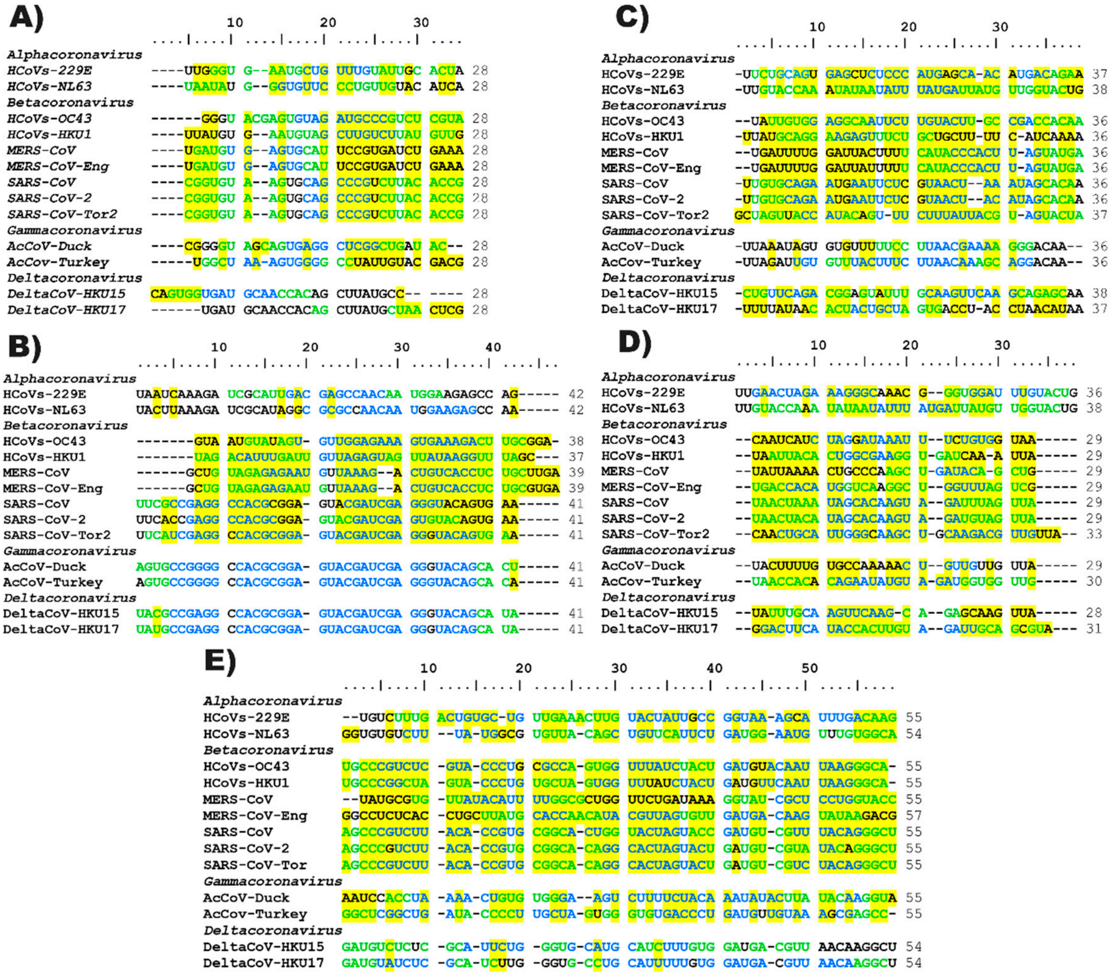

Figure S1. Sequence alignment of cis elements in alpha, beta, gamma and deltacoronavirus. A) Stem-loop1; B) s2m; C) Psedoknot stem-loop1; D) Pseudoknot stem-loop2; E) Stem-loop2. Alphacoronavirus: HCoVs-229E (NP_073549.1), HCoVs-NL63 (AVA2672.1); Betacoronavirus: HCoVs-OC43 (YP_009555238.1), HCoVs-HKU1 (YP_173236.1), MERS-CoV (YP_009047202.1), MERS-CoV-Eng (YP_007188577.3), SARS-CoV (APO40578.1), SARS -CoV-2 (YP_009724389.1), SARS-CoV-Tor2 (NP_828849.7); Gammacoronavirus: AcCoV-Duck (YP_009825006.1), AcCoV-Turkey (YP_001941164.2); Deltacoronavirus: Porcine coronavirus HKU15 (QWE80491.1), Sparrow deltacoronavirus HKU17 (AWV67106.1).

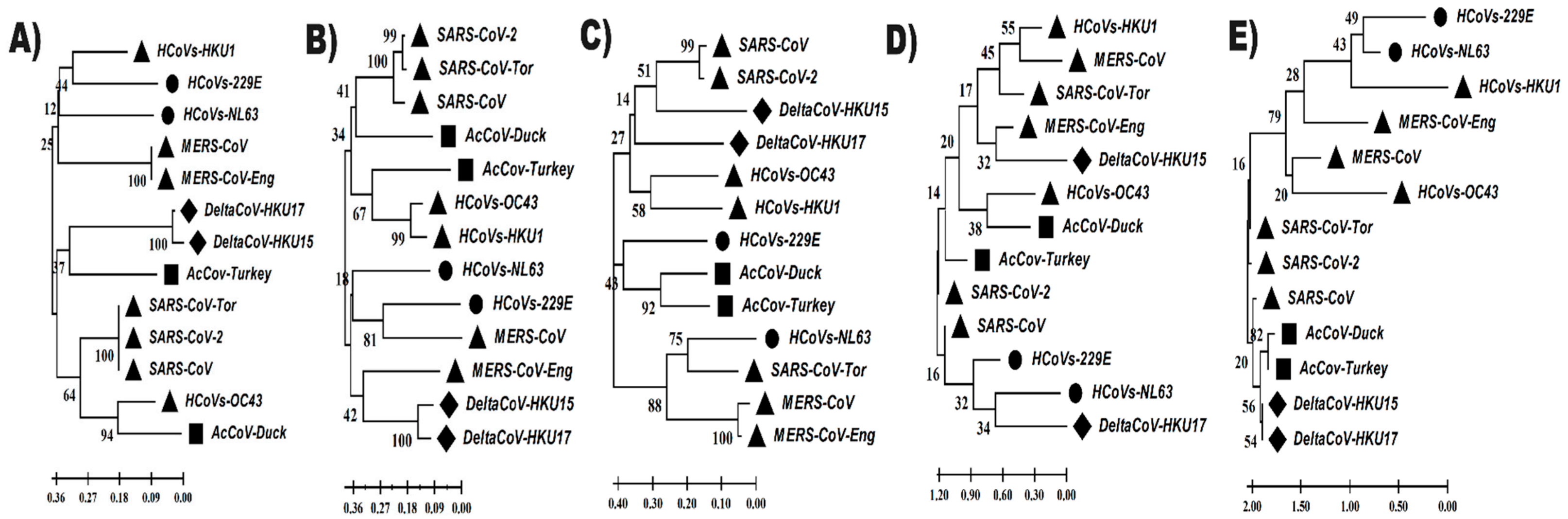

Figure S2. Evolutionary relationships of cis elements in human pathogenic coronaviruses.

Tables

Table S1. Evolutionarily conserved RNA secondary structures of stem-lopp1 of coronavirus.

**Coronavirus**	**ΔG (kcal/mol)**	**z-score***	**p-value****
	Alphacoronavirus		
HCoVs-229E	-7.57	-0.862	0.81
HCoVs-NL63	-5.62	0.659	0.12
	Betacoronavirus		
HCoVs-OC43	-6.58	-0.061	0.43
HCoVs-HKU1	-1.40	0.557	0.23
HCoVs			0.33
MERS-CoV	-2.38	-1.449	0.98
MERS-CoV-Eng	-2.37	-1.328	1.00
MERS			0.99
SARS-CoV	-12.91	-0.744	0.76
SARS-CoV-2	-12.93	-0.641	0.76
SARS-CoV-Tor2	-12.93	-0.735	0.75
	Gammacoronavirus		
AcCoV-Duck	-5.28	-0.613	0.84
AcCov-Turkey	-4.67	0.210	0.34
	Deltacoronavirus		
DeltaCoV-HKU15	-8.45	1.738	0.08
DeltaCoV-HKU17	-2.49	1.363	0.10

*Method SCI based on minimum free energies.
**Empirical probability of evolutionarily conserved RNA secondary structures.

Table S2. Evolutionarily conserved RNA secondary structures of stem-lopp2 of coronavirus.

Coronavirus	ΔG (kcal/mol)	z-score*	p-value**
	Alphacoronavirus		
HCoVs-229E	-9.08	-0.870	0.89
HCoVs-NL63	-13.06	0.821	0.13
	Betacoronavirus		
HCoVs-OC43	-16.58	-0.093	0.49
HCoVs-HKU1	-17.37	0.417	0.19
HCoVs			0.34
MERS-CoV	-11.34	0.906	0.24
MERS-CoV-Eng	-10.46	-1.054	0.96
MERS			0.60
SARS-CoV	-19.00	-0.899	0.79
SARS-CoV-2	-19.76	-0.739	0.76
SARS-CoV-Tor2	-16.69	-0.849	0.81
	Gammacoronavirus		
AcCoV-Duck	-8.53	-0.425	0.80
AcCov-Turkey	-18.56	0.020	0.49
	Deltacoronavirus		
DeltaCoV-HKU15	-13.30	1.682	0.05
DeltaCoV-HKU17	-13.22	1.550	0.07

*Method SCI based on minimum free energies.

** Empirical probability of evolutionarily conserved RNA secondary structures.

Table S3. Evolutionarily conserved RNA secondary structures of Pseudoknot stem-lopp1 of coronavirus.

Coronavirus	ΔG(kcal/mol)	z-score*	p-value**
	Alphacoronavirus		
HCoVs-229E	-13.08	-0.440	0.63
HCoVs-NL63	-11.47	0.175	0.97
	Betacoronavirus		
HCoVs-OC43	-13.34	0.191	0.30
HCoVs-HKU1	-7.28	-0.513	1.00
HCoVs			0.65
MERS-CoV	-4.18	0.889	0.06
MERS-CoV-Eng	-4.21	-0.250	0.44
MERS			0.25
SARS-CoV	-6.09	0.174	0.14
SARS-CoV-2	-6.27	0.079	0.31
SARS-CoV-Tor2	-2.21	-0.412	0.53
	Gammacoronavirus		
AcCoV-Duck	-5.24	-0.269	0.77
AcCov-Turkey	-3.45	-0.273	0.75
	Deltacoronavirus		
DeltaCoV-HKU15	-6.47	-0.155	0.38
DeltaCoV-HKU17	-4.69	-0.352	0.86

*Method SCI based on minimum free energies.
**Empirical probability of evolutionarily conserved RNA secondary structures.

Table S4. Evolutionarily conserved RNA secondary structures of Pseudoknot stem-lopp2 of coronavirus.

Coronavirus	ΔG(kcal/mol)	z-score*	p-value**
	Alphacoronavirus		
HCoVs-229E	-4.20	-0.375	0.52
HCoVs-NL63	-11.47	0.251	0.94
	Betacoronavirus		
HCoVs-OC43	-4.44	-0.011	0.20
HCoVs-HKU1	-3.66	-0.799	1.00
HCoVs			0.60
MERS-CoV	-5.01	2.116	0.04
MERS-CoV-Eng	-9.19	-0.197	0.47
MERS			0.51
SARS-CoV	-6.33	0.134	0.20
SARS-CoV-2	-8.82	0.179	0.20
SARS-CoV-Tor2	-5.44	-0.211	0.46
	Gammacoronavirus		
AcCoV-Duck	-1.47	-0.651	0.82
AcCov-Turkey	-7.98	-0.316	0.68
	Deltacoronavirus		
DeltaCoV-HKU15	-4.32	-0.117	0.38
DeltaCoV-HKU17	-2.66	-0.561	0.80

*Method SCI based on minimum free energies.

** Empirical probability of evolutionarily conserved RNA secondary structures.

Table S5. Evolutionarily conserved RNA secondary structures of s2m of coronavirus.

Coronavirus	ΔG(kcal/mol)	z-score*	p-value**
Alphacoronavirus			
HCoVs-229E	-3.10	-0.986	0.81
HCoVs-NL63	-6.70	0.799	0.14
Betacoronavirus			
HCoVs-OC43	-2.90	-0.050	0.47
HCoVs-HKU1	3.70	0.536	0.22
HCoVs			0.34
MERS-CoV	-8.40	-0.816	0.75
MERS-CoV-Eng	-8.90	-0.830	0.78
MERS			0.76
SARS-CoV	-7.70	-0.883	0.81
SARS-CoV-2	-6.10	-0.775	0.77
SARS-CoV-Tor2	-6.60	-0.897	0.82
Gammacoronavirus			
AcCoV-Duck	-10.20	-0.474	0.74
AcCov-Turkey	-10.10	-0.458	0.72
Deltacoronavirus			
DeltaCoV-HKU15	-8.30	-1.610	0.75
DeltaCoV-HKU17	-8.30	-0.998	0.73

*Method SCI based on minimum free energies.
**Empirical probability of evolutionarily conserved RNA secondary structures.

Table S6. Evolutionarily conserved RNA secondary structures of stem-loop unit of coronavirus.

Coronavirus	ΔG(kcal/mol)	z-score*	p-value**
	Alphacoronavirus		
HCoVs-229E	-18.15	0.000	1.00
HCoVs-NL63	-19.47	-1.075	0.92
	Betacoronavirus		
HCoVs-OC43	-32.85	0.000	1.00
HCoVs-HKU1	-19.09	0.000	1.00
HCoVs			1.00
MERS-CoV	-18.81	0.000	1.00
MERS-CoV-Eng	-17.32	0.000	1.00
MERS			1.00
SARS-CoV	-33.21	0.000	1.00
SARS-CoV-2	-33.97	0.000	1.00
SARS-CoV-Tor2	-30.90	0.000	1.00
	Gammacoronavirus		
AcCoV-Duck	-21.44	-0.668	0.76
AcCov-Turkey	-27.22	0.000	1.00
	Deltacoronavirus		
DeltaCoV-HKU15	-25.32	0.427	0.29
DeltaCoV-HKU17	-25.95	1.218	0.12

*Method SCI based on minimum free energies.
**Empirical probability of evolutionarily conserved RNA secondary structures.

Table S7. Evolutionarily conserved RNA secondary structures of pseudoknot stem-loop unit of coronavirus.

Coronavirus	ΔG(kcal/mol)	z-score*	p-value**
	Alphacoronavirus		
HCoVs-229E	-17.61	0.000	1.00
HCoVs-NL63	-26.91	0.000	1.00
	Betacoronavirus		
HCoVs-OC43	-17.97	0.000	1.00
HCoVs-HKU1	-11.30	0.000	1.00
HCoVs			1.00
MERS-CoV	-10.52	0.000	1.00
MERS-CoV-Eng	-14.07	0.000	1.00
MERS			1.00
SARS-CoV	-12.95	0.000	1.00
SARS-CoV-2	-15.62	0.000	1.00
SARS-CoV-Tor2	-12.72	0.000	1.00
	Gammacoronavirus		
AcCoV-Duck	-12.08	0.000	1.00
AcCov-Turkey	-12.24	0.000	1.00
	Deltacoronavirus		
DeltaCoV-HKU15	-14.56	0.000	1.00
DeltaCoV-HKU17	-8.94	0.000	1.00

*Method SCI based on minimum free energies.

**Empirical probability of evolutionarily conserved RNA secondary structures.

Table S8. Changes in stem-loop1 ribonucleotides of Coronavirus variants.

Coronavirus	Percentage of changes*	Ribonucleotides (position)**
		Alphacoronavirus
HCoVs-229E	46% (15rb)	U(6), G(7), G(8), G(9), A(14), A(15), C(18), G(20), U(21), U(22), A(26), U(27), G(29), C(32), U(33).
HCoVs-NL63		A(6), A(7), U(8), A(9), G(14), G(15), U(18), C(20), C(21), C(22), U(26), G(27), A(29), U(32), C(33).
		Betacoronavirus
HCoV-OC43	64% (18rb)	G(7), G(8), A(11), G(15),G(20), A(21), U(22), G(23), C(24), C(25), C(26), G(27), C(29), U(30), C(31), G(32), U(33), A(34).
HCoV-HKU1	71% (20rb)	U(5), U(6), A(7), U(8), G(11), A(15), G(20), C(21), U(22), U(23), G(24), U(25), C(26), U(27), A(29), U(30), G(31), U(32), U(33), G(34).
MERS-CoV		U(5), U(6), A(7), U(8), G(11), G(15), U(20), U(21), C(22), C(23), G(24), U(25), G(26), A(27), C(29), U(30), G(31), A(32), A(33), A(34).
MERS-Cov-Eng		
SARS-CoV	71% (20rb)	C(5), G(6), G(7), U(8), A(11), G(15), G(20), C(21), C(22), C(23), G(24), U(25), C(26), U(27), A(29), C(30), A(31), C(32), C(33), G(34).
SARS-CoV-2		
SARS-CoV-Tor2		
		Gammacoronavirus
AcCoV-Duck	57% (16rb)	C(5), G(6), G(9), G(12), C(13), A(18), U(22),C(23), G(24), G(25), C(26),U(27), G(28), U(30), A(31), C(32).
AcCoV-Turkey		U(6), C(9), A(12), G(18), C(22), U(23), A(24), U(25), U(26), G(27), U(28), C(30), G(31), A(32), C(33), G(34).
		Deltacoronavirus
DeltaCoV-HKU15	25% (7rb)	C(1), A(2), G(3), U(4), G(5), G(6), C(29).
DeltaCoV-HKU17		U(28), A(29), A(30), C(31), U(32), C(33), G(34).

**Regarding the total number of ribonucleotides of 28 of each cis element.*

***Regarding the position of each ribonucleotide in the sequence alignment (See Figure S1).*

Table S9. Changes in stem-loop2 ribonucleotides of Coronavirus variants

Coronavirus	Percentage of changes *	Ribonucleotides (position)**
		Alphacoronavirus
HCoVs-229E	62% (34rb)	C(6), U(8), G(10), A(11), C(12), G(14), U(15), G(16), C(17),U(18), U(22), G(23), A(24),A(26), U(28), U(29), G(30), A(32), C(33), A(35), U(36), U(38), G(38), C(40), G(41), A(44), A(45), G(48), C(49), A(50), A(55), C(56), A(57), A(58), G(59).
HCoVs-NL63	61% (33rb)	G(1), G(2), G(6), C(8), U(10), A(14), U(16), G(17), G(18), C(19), G(22), U(23), U(24), A(28), G(29), C(30), G(32), U(33), C(35), A(36), U(38), U(40), A(41), G(44), G(45), A(48), U(49), G(50), U(55), G(56), G(57), C(58), A(59).
		Betacoronavirus
HCoV-OC43	89% (49rb)	U(1), C(3), C(4), C(5), G(6), U(7), C(8), U(9), C(10), G(12), U(13), A(14), C(16), C(17), C(18), G(20), C(21), G(22), C(23), C(24), A(25), G(27), U(28), G(29), G(30), U(31), U(32), U(33), A(34), U(35), C(36), A(38), C(39), U(40), A(42), G(44), U(45), A(46), A(48), A(49), U(50), U(51), A(52), A(53), G(54), G(55), G(56), C(57), A(58).
HCoV-HKU1		U(1), C(3), C(4), C(5), G(6), G(7), C(8), U(9), A(10), G(12), U(13), A(14), C(16), C(17), C(18), G(20), U(21), G(22), C(23), U(24), A(25), G(27), U(28), G(29), G(30), U(31), U(32), U(33), A(34), U(35), C(36), A(38), C(39), U(40), A(42), G(44), U(45), U(46), A(48), A(49), U(50), U(51), A(52), A(53), G(54), G(55), G(56), C(57), A(58).
MERS-CoV	91%(51rb)	U(3), A(4), U(5), G(6), C(7), G(8), U(9), G(10), U(12), U(13), A(14), U(15), A(16), C(17), A(18), U(20), U(21), U(22), G(23), G(24), C(25), G(26), C(27), U(28), G(29), G(30), U(31), U(32), C(33), U(34), G(35), A(36), A(38), A(39), A(40), G(42), A(44), U(45), G(48), C(49), U(50), C(51), C(52), U(53), G(54), G(55), U(56), A(57), C(58), C(59).
MERS-Cov-Eng	89%(57rb)	G(1), C(3), C(4), U(5), C(6), U(7), C(8), A(9), C(10), C(12), U(13), G(14), C(15), U(16), U(17), A(18), G(20), C(21), A(22), C(23), C(24), A(25), A(26), C(27), A(28), U(29), A(30), C(31), G(32), U(33), U(34), A(35), G(36), G(38), U(39), U(40), A(42), G(44), A(45), A(48), A(49), G(50), U(51), A(52), U(53), A(54), A(55), G(56), A(57), C(58), G(59).
SARS-CoV	89%(49rb)	A(1), C(3), C(4), C(5), G(6), U(7), C(8), U(9), U(10), A(12), C(13), A(14), C(16), C(17), G(18), G(20), C(21), G(22), G(23), C(24), A(25) C(27), U(28), G(29), G(30), U(31), A(32), C(33), U(34), A(35), G(36), A(38), C(39), C(40), A(42), G(44), U(45), G(48), U(49), U(50), U(51), A(52), C(53), A(54), G(55), G(56), G(57), C(58), U(59).
SARS-CoV-2		A(1), C(3), C(4), C(5), G(6), U(7), C(8), U(9), U(10), A(12), C(13), A(14), C(16), C(17), G(18), G(20), C(21), G(22), G(23), C(24), A(25) C(27), A(28), G(29), G(30), C(31), A(32), C(33),

		U(34), A(35), G(36), A(38), C(39), U(40), A(42), G(44), U(45), G(48), U(49), **A(50),** U(51), A(52), C(53), A(54), G(55), G(56), G(57), C(58), U(59).
SARS-CoV-Tor2		A(1), C(3), C(4), C(5), G(6), U(7), C(8), U(9), U(10), A(12), C(13), A(14), C(16), C(17), G(18), G(20), C(21), G(22), G(23), C(24), A(25) C(27), A(28), G(29), G(30), C(31), A(32), C(33), U(34), A(35), G(36), A(38), C(39), U(40), A(42), G(44), U(45), G(48), U(49), C**(50),** U(51), A(52), C(53), A(54), G(55), G(56), G(57), C(58), U(59).
	Gammacoronavirus	
AcCoV-Duck	60%(33rb)	A(1), A(2), U(3), C(4), A(6), C(7), A(10), A(13), G(18), U(19), G(20), G(23), G(24), A(28), U(30), C(31), U(33), U(35), C(36), U(37), A(38), A(40), A(41), A(44), A(46), C(47), U(49), U(51), A(52), A(54), G(57), U(58), A(59).
AcCoV-Turkey		G(1), G(2), C(3), U(4), G(6), G(7), G(10), U(13), C(18), C(19), U(20), C(23), U(24), G(27), U(28), G(30), G(31), G(33), G(35), A(36), C(37), C(38), U(40), G(41), G(45), U(46), G(47), A(49), A(51), G(52), G(54), C(57), C(58).
	Deltacoronavirus	
DeltaCoV-HKU15	9%(5rb)	C(6), U(17), C(18), A(28), C(34)
DeltaCoV-HKU17		A(6), C(17), U (18), C(28), U(34)

**Regarding the ribonucleotide length of 55 rb of each cis element, except for HCoVs-NL63 (54rb), MERS-CoV-Eng (57rb), DeltaCoV-HKU15 and -HKU17 (54rb).*

***Regarding the position of each ribonucleotide in the sequence alignment (See Figure S1).*

Table S10. Changes in Pseudoknot stem-loop1 ribonucleotides of Coronavirus variants

Coronavirus	Percentage of changes *	Ribonucleotides (position)**
		Alphacoronavirus
HCoVs-229E	78% (29rb)	C(4), G(6), A(8), G(9), U(10), G(11), A(12), G(13), C(14), U(15), C(16), C(18), C(19), C(20), A(21), U(22), G(23), A(24), G(25), C(26), A(27), A(29), C(30), A(31), A(34), C(35), G(37), A(38), A(39)
HCoVs-NL63	79% (30rb)	G(4), A(6), C(8), A(9), A(10), A(11), U(12), A(13), U(14), A(15), A(16), A(18), U(19), U(20), U(21), A(22), U(23), G(24), A(25), U(26), U(27), A(28), U(29), G(30), U(31), G(34), U(35), C(37), U(38), G(39)
		Betacoronavirus
HCoV-OC43	86% (31rb)	A(4), U(5), U(6), G(7), U(8), G(9), G(10), A(11), G(12), G(13), C(14), A(15), A(16), U(18), C(19), U(20), U(21), G(22), U(23), A(24), C(25), U(26), U(27), G(29), C(30), C(31), G(32), C(34), C(35), C(37), A(38)
HCoV-HKU1		U(2), A(4), U(5), G(6), C(7), A(8), G(9), G(10), A(11), A(12), G(13), A(14), G(15), U(16), U(18), C(19), U(20), G(21), C(22), U(23), G(24), C(25), U(26), U(27), U(29), U(30), C(31), U(34), C(35), A(37), A(38)
MERS-CoV	89% (32rb)	G(4), A(5), U(6), U(7), U(8), U(9), G(10), G(11), A(12), U(13), U(14), A(15), C(16), U(18), U(19), U(20), C(21), A(22), U(23), A(24), C(25), C(26), C(27), A(28), C(29), U(30), U(31), G(34), U(35), U(37), G(38)
MERS-Cov-Eng		G(4), A(5), U(6), U(7), U(8), U(9), G(10), G(11), A(12), U(13), U(14), A(15), U(16), U(18), U(19), U(20), C(21), A(22), U(23), A(24), C(25), C(26), C(27), A(28), C(29), U(30), U(31), G(34), U(35), U(37), G(38)
SARS-CoV	86% (31rb)	U(2), G(4), U(5), G(6), C(7), A(8), G(9), A(10), A(11), U(12), G(13), A(14), A(15), U(16), C(18), U(19), C(20), G(21), U(22), A(23), A(24), C(25), U(26), A(29), A(30), A(31), U(32), G(34), C(35), C(37), A(38)
SARS-CoV-2		U(2), G(4), U(5), G(6), C(7), A(8), G(9), A(10), A(11), U(12), G(13), A(14), A(15), U(16), C(18), U(19), C(20), G(21), U(22), A(23), A(24), C(25), U(26), A(29), C(30), A(31), U(32), G(34), C(35), C(37), A(38)
SARS-CoV-Tor2	86% (32rb)	G(1), C(2), A(4), G(5), U(6), U(7), A(8), C(9), C(10), A(11), U(12), A(13), C(14), A(15), G(16), U(19), U(20), C(21), U(22), U(23), U(24), A(25), U(26), U(27), A(28), C(29), G(30), U(31), G(34), U(35), C(37), U(38)
		Gammacoronavirus
AcCoV-Duck	28% (10rb)	A(5), A(8), G(13), U(15), U(16), C(19), G(26), A(29), A(30), G(31)
AcCoV-Turkey		G(5), U(8), U(13), A(15), C(16), U(19), A(26), G(29), C(30), A(31)

	Deltacoronavirus	
DeltaCoV-HKU15	68% (26rb)	C(2), U(3), G(4), U(6), C(7), G(9), A(10), C(11), G(12), G(13), G(15), A(17), U(18), U(20), C(22), A(23), G(25), U(26), C(28), A(30), G(31), C(32), G(34), A(35), G(36), C(37)
DeltaCoV-HKU17	68%(25rb)	U(2), U(3), U(4), A(6), U(7), A(9), C(10), A(11), C(12), U(13), C(15), G(17), C(18), A(20), U(22), G(23), C(24), C(25), C(30), C(32), U(32), A(34), C(35), A(36), U(37)

**Regarding the ribonucleotide length of 36 rb of each cis element, except for HCoVs-229E (37rb), SARS-CoV-Tor2 (37rb), DeltaCoV-HKU15 (38rb) and -HKU17 (37rb).*

***Regarding the position of each ribonucleotide in the sequence alignment (See Figure S1).*

Table S11. Changes in Pseudoknot stem-loop2 ribonucleotides of Coronavirus variants

Coronavirus	Percentage of changes*	Ribonucleotides (position)**
		Alphacoronavirus
HCoVs-229E	47% (17rb)	A(4), G(9), A(11), G(13), G(14), G(15), C(16), A(18), A(19), C(20), G(21), G(24), G(25), G(27), G(28), A(29), U(32)
HCoVs-NL63	47% (18rb)	U(4), A(9), U(11), U(13), A(14), A(15), U(16), U(18), U(19), U(20), U(22), G(23), A(24), U(25), A(27), U(28), G(29), G(32)
		Betacoronavirus
HCoV-OC43	93% (27rb)	C(3), A(4), A(5), U(6), C(7), A(8), U(9), C(10), U(11), A(12), G(14), A(15), U(16), A(18), A(19), U(20), U(21), U(24), C(25), U(26), G(27), U(28), G(29), G(30), U(31), A(32), A(33)
HCoV-HKU1	90% (26rb)	U(3), A(4), A(5), U(6), U(7), A(8), C(9), A(10), C(11), U(12), G(14), C(15), G(16), A(18), G(19), G(20), U(21), A(24), U(25), C(26), A(27), A(28), A(30), U(31), U(32), A(33)
MERS-CoV		U(3), A(4), U(5), U(6), A(7), A(8), A(9), A(10), C(11), U(12), C(14), C(15), C(16), A(18), G(19), C(20), U(21), A(24), U(25), A(26), C(27), A(28), G(30), C(31), U(32), G(33)
MERS-Cov-Eng	93% (27rb)	U(3), G(4), A(5), C(6), C(7), A(8), C(9), A(10), U(11), G(12), U(14), C(15), A(16), G(18), G(19), C(20), U(21), G(24), G(25), U(26), U(27), U(28), A(29), G(30), U(31), C(32), G(33)
SARS-CoV		U(3), A(4), A(5), C(6), U(7), A(8), A(9), A(10), U(11), A(12), C(14), A(15), C(16), A(18), G(19), U(20), A(21), G(24), A(25), U(26), U(27), U(28), A(29), G(30), U(31), U(32), A(33)
SARS-CoV-2		U(3), A(4), A(5), C(6), U(7), A(8), A(9), A(10), U(11), A(12), C(14), A(15), C(16), A(18), G(19), U(20), A(21), G(24), A(25), U(26), **G**(27), U(28), A(29), G(30), U(31), U(32), A(33)
SARS-CoV-Tor2	91% (30rb)	C(3), A(4), A(5), C(6), U(7), G(8), C(9), A(10), U(11), U(12), G(14), G(15), C(16), A(18), G(19), C(20), U(21), C(24), A(25), A(26), G(27), A(28), C(29), G(30), U(31), U(32), G(33), U(34), U(35), A(36)
		Gammacoronavirus
AcCoV-Duck	66% (19rb)	C(5), U(6), U(7), U(8), U(9), G(10), U(11), G(12), C(13), C(14), A(16), A(18), A(19), C(20), U(21), G(24), U(26), U(29), A(33)
AcCoV-Turkey	67% (20rb)	A(5), C(6), C(7), A(8), C(9), A(10), C(11), A(12), G(13), A(14), U(16), U(18), G(19), U(20), A(21), G(23), A(24), G(26), G(29), G(33)
		Deltacoronavirus
DeltaCoV-HKU15	79% (22rb)	U(3), A(4), U(5), U(6), G(8), A(11), G(12), U(13), U(14), C(15), A(16), A(17), G(18), C(20), G(26), C(27), A(28), A(29), G(30), U(31), U(32), A(33)
DeltaCoV-HKU17	81% (25rb)	G(3), G(4), A(5), C(6), U(8), U(11), A(12), C(13), C(14), A(15), C(16), U(17), U(18), G(19), U(20), U(26), U(27), G(28), C(29), A(30), G(31), C(32), G(33), U(34), A(35)

**Regarding the ribonucleotide length of each cis element. In the variants HCoVs-OC43, HCoVs-HKU1, MERS-CoV, MERS-CoV-Eng, SARS-CoV, SARS-CoV-2 it is 29rb.And for HCoVs-229E,*

HCoVs-NL63, SARS-CoV-Tor2, AcCoV-Duck, AcCoV-Turkey, DeltaCoV-HKU15 and DeltaCoV-HKU17 is 36, 38, 33, 29, 30, 28, 31 rb respectively.

***Regarding the position of each ribonucleotide in the sequence alignment (See Figure S1).*

Table S12. Changes in s2m ribonucleotides of Coronavirus variants

Coronavirus	Porcentaje de cambios*	Ribonucleótidos (posición)**
		Alphacoronavirus
HCoVs-229E	14% (6rb)	A(3), C(5), U(7), A(9), A(22), G(42)
HCoVs-NL63	14% (6rb)	C(3), U(5), A(7), G(9), C(22), A(42)
		Betacoronavirus
HCoV-OC43	95% (36rb)	G(8), U(9), A(10), A(11), U(12), G(13), U(14), A(15), U(16), A(17), G(18), U(19), U(23), G(24), G(25), A(26), G(27), A(28), A(29), A(30), G(31), U(32), G(33), A(34), A(35), A(36), G(37), A(38), C(39), U(40), U(41), G(42), C(43), G(44), G(45), A(46)
HCoV-HKU1	95%(35rb)	U(8), A(9), G(10), A(11), C(12), A(13), U(14), U(15), U(16), G(17), A(18), U(19), U(20), U(23), A(24), G(25), A(26), G(27), U(28), A(29), G(30), U(31), U(32), A(33), U(34), A(35), A(36), G(37), G(38), U(39), U(40), U(41), A(42), G(43), C(44)
MERS-CoV	92%(36rb)	C(8), U(9), G(10), U(11), A(12), G(13), A(14), G(15), A(16), G(17), A(18), A(19), U(20), U(23), A(24), A(25), A(26), G(27), A(30), C(31), U(32), G(33), U(34), C(35), A(36), C(37), C(38), U(39), C(40), U(41), G(42), C(43), U(44), U(45, G(46), A(47)
MERS-Cov-Eng		C(8), U(9), G(10), U(11), A(12), G(13), A(14), G(15), A(16), G(17), A(18), A(19), U(20), U(23), A(24), A(25), A(26), G(27), A(30), C(31), U(32), G(33), U(34), C(35), A(36), C(37), C(38), U(39), C(40), U(41), G(42), C(43), G(44), U(45), G(46), A(47)
SARS-CoV	83%(34rb)	G(4), C(5), A(8), G(9), G(10), C(11), C(12), A(13), C(14), G(15), C(16), G(17), G(18), A(19), A(23), C(24), G(25), A(26), U(27), C(28), G(29), A(30), G(31), G(32), G(33), U(34), A(35), C(36), A(37), G(38), U(39), G(40), A(41), A(42)
SARS-CoV-2		A(4), C(5), A(8), G(9), G(10) C(11), C(12), A(13), C(14), G(15), C(16), G(17), G(18), A(19), A(23), C(24), G(25), A(26), U(27), C(28), G(29), A(30), G(31), U(32), G(33), U(34), A(35), C(36), A(37), G(38), U(39), G(40), A(41), A(42)
SARS-CoV-Tor2		A(4), U(5), A(8), G(9), G(10) C(11), C(12), A(13), C(14), G(15), C(16), G(17), G(18), A(19), A(23), C(24), G(25), A(26), U(27), C(28), G(29), A(30), G(31), G(32), G(33), U(34), A(35), C(36), A(37), G(38), U(39), G(40), A(41), A(42)
		Gammacoronavirus
AcCoV-Duck	2%(1rb)	U(42)
AcCoV-Turkey		A(42)
		Deltacoronavirus
DeltaCoV-HKU15	2%(1rb)	C(3)
DeltaCoV-HKU17		U(3)

**Regarding the ribonucleotide length of each cis element: HCoVs-229E, HCoVs-NL63 was 42rb and SARS-CoV, SARS-CoV-2, SARS-CoV-Tor2 is 41rb. In the HCoVs-OC43, HCoVs-HKU1, MERS-CoV, MERS-CoV-Eng variants it is 38, 37 and 39 rb, respectively.*

***Regarding the position of each ribonucleotide in the sequence alignment (See Figure S1).*
